# Genome-wide dissection of AT-hook motif nuclear-localized gene family and their expression profiling for drought and salt stress in rice (*Oryza sativa*)

**DOI:** 10.3389/fpls.2023.1283555

**Published:** 2023-12-12

**Authors:** Dhanorkar A. Ambadas, Ashutosh Singh, Ratnesh Kumar Jha, Divya Chauhan, Santhosh B., Vinay Kumar Sharma

**Affiliations:** ^1^ Department of AB&MB, CBSH, Dr. Rajendra Prasad Central Agricultural University, Samastipur, Bihar, India; ^2^ Centre for Advanced Studies on Climate Change, Dr. Rajendra Prasad Central Agricultural University, Samastipur, Bihar, India; ^3^ Department of Bioscience and Biotechnology, Banasthali Vidyapith, Aliyabad, Rajasthan, India

**Keywords:** AT-hook gene family, rice development, transcriptome, co-expression network analysis, drought and salt stress, gene expression

## Abstract

AT-hook motif nuclear localized (*AHL*) genes are functionally very less explored, but their nature is very diverse. In the present study, we identified 20 *AHL* genes in rice. Phylogenetic analyses and evolutionary classification of *AHL* genes showed that they are conserved in plants, but the number of genes is still expanding in different crops and regulating new biological functions. Gene structure analysis showed that *OsAHL*s are with and without intron types of genes, suggesting that *AHL* genes added intron during evolution for neofunctionalization. The *cis* analysis of *OsAHL* genes suggested its motif diversity. In order to understand the function, 19 transcriptomes were identified from various tissues and different developmental stages of rice, and they were divided into eight groups by different temporal and spatial expression. Through co-expression analysis, 11 Os*AHLs* and 13 novel genes with intricate networks that control many biological pathways in rice were identified. The interactions of OsAHL proteins showed that they co-regulate important processes including flowering, reproductive organ development, and photosynthesis activity. The functionality of all 20 genes of *OsAHL* for drought and salt stress in leaf tissues of two contrasting genotypes (IR64 and NL44) of rice was studied using qRT-PCR. The result clearly showed significant upregulation of *OsAHL* genes under drought and salt conditions over the control. The differential expression between IR64 and NL44 showed a significant upregulation of *OsAHL* genes in NL44 as compared to the IR64 genotype under drought and salt stress. Overall, the result indicates that AHL genes might be involved in mediating drought and salt-signaling transduction pathways. The drought- and salt-tolerant nature of NL44 was also confirmed by expression profiling.

## Introduction

Rice is the world’s most important crop in terms of both land area and production (Chauhan et al., 2017). Due to diverse growing conditions, rice is exposed to different environmental stresses. Rice yield is influenced by biotic and abiotic stresses (Anami et al., 2020). Among abiotic stresses, drought, heat, and salinity contribute the most to yield loss (Pathak et al., 2021). Drought and salinity stresses affect rice growth and productivity significantly (Radha et al., 2023). Drought stress causes a 13%–35% yield loss of rice with an area of approximately 42 mha per year grown under rainfed upland and lowland areas (Shamsudin et al., 2016). Therefore, it is very important to understand the patterns of response in rice against different abiotic stresses. The rice crop has evolved with various molecular mechanisms of different levels, such as morphological, cellular, physiological, and molecular levels, in response to abiotic stresses (Zhu, 2016). Rice crops developed a complex signaling network at molecular and cellular levels to tackle drought stress. The gene expression is controlled by transcription factors (TFs), and TFs regulate the drought stress response. They control genes in a synchronized manner. Therefore, it is an attractive target, and its characterization by molecular biology tools has a very important application in crop improvement. There are different TFs characterized and utilized for crop improvement. The AT-hook motif nuclear-localized (AHL) family of TFs is highly conserved in all crop plants. While the conservation nature of AHL in all land plants indicates its importance, we have just begun to understand its role in growth, development, and stress tolerance processes (Zhu, 2002). Family members of AHLs in *Hordeum vulgare* have shown diverse roles in development (Tripathi et al., 2018). Apart from growth and development, AHLs have also been playing an active role in drought tolerance (Zhou et al., 2016; Wong et al., 2019) and plant immunity (Lu et al., 2010) resistance against ascochyta blight (Kumar et al., 2018).

The AHL proteins consist of an AT-Hook (AH) motif and conserved domain of Plants and Prokaryotes Conserved (PPC); it is conserved from plant to prokaryotic system and is also called Domain of Unknown Function 296 (DUF 296) (Zhao et al., 2014). It is a non-histone chromosomal protein high-mobility group (HMG)-1/Y (High-mobility group) of a high-mobility group that first revealed the AHL (HMG) small DNA binding protein motif. The AHL is a DNA binding protein motif with non-histone chromosomal protein HMG-1/Y of the HMG. By creating homo/hetero-trimeric DNA–protein and protein–protein complexes, the *AHL* family of a gene regulates plant growth and development (Huth et al., 1997; Zhao et al., 2013). The *AHL* family has conserved AT-hook and PPC/DUF domain structures. The *AHL* gene family members have differences in terms of gene size, gene organization, motif number, and AT-hook and PPC motif sequences (Kim et al., 2011). There are some reports in model plants such as *Arabidopsis* and rice such as *OsAHL1* gene, which enhances drought resistance in rice by regulating drought-related genes during the panicle stage of development. Meanwhile, the overexpression of *OsAHL1* improved drought and cold tolerance (Zhou et al., 2016). Despite such diverse roles, there is a lack of comprehensive understanding of its functional diversity and sequence information. The abovementioned literature showed that limited information has been generated in model plant *Arabidopsis* on the different roles of *AHL* genes, but the information/role related to the grass family is very limited is quite striking.

Therefore, the purpose of the present study was evolutionary analysis, protein interaction, tissue-specific expression, and validation of drought and salinity stresses in contrasting genotypes of rice. This study improves the knowledge of diversity and novel gene and their interaction with *AHL* genes in plant development and provides a way for targeting the *AHL* gene family for increasing productivity and climate-resilient crops.

## Materials and methods

### Materials

Genotypes IR64 and NL44 of rice were collected from the physiology division of the Centre of Advanced Studies on Climate Change, Dr. Rajendra Prasad Central Agricultural University, Pusa, Samastipur, Bihar-848125 (India).

### Genome-wide identification of AHL proteins

The AHL TF family members were identified from the genome of *Oryza sativa* L. *japonica* (cultivar Nipponbare) by retrieving the data from Ensembl plants (https://plants.ensembl.org/Oryza_sativa/Info/Index) (Contreras-Moreira et al., 2022). The complete rice proteome was scanned using the hidden Markov model (HMM) for the PPC/DUF296 (PF03479) domain, and Pfam ID was obtained using the Pfam database (https://pfam.xfam.org/) (El-Gebali et al., 2019). The domain characteristics were confirmed by searching retrieved sequences against the Conserved Domains Database (CDD) (www.ncbi.nlm.nih.gov/Structure/cdd/wrpsb.cgi) using 10^−5^ of e-value. Sequences consisting of a PPC domain with an AT-hook motif were considered as a group of AHL transcription factors. These sequences were searched against the Rice Genome Annotation Project (RGAP) database for gene annotation (e-value 10^−5^). Further, RGAP was used for gene ontology mapping (http://rice.uga.edu/).

### Mapping, gene structure, and *cis*-element analysis

All 20 *AHL* family genes were mapped to 12 rice chromosomes using MapChart software (https://www.wur.nl/en/show/mapchart.htm). The alignment of exons and introns was determined by aligning the coding sequences with genomic sequences obtained from Ensembl plants (http://plants.ensembl.org/index.html). *Cis*-elements of *AHL* family genes of rice were identified by sequences located 5′ UTR of 2 kb upstream of transcription start sites that are considered promoters. The promoter sequence AHL family genes were downloaded from the Ensembl rice biomart database (http://plants.ensembl.org/biomart/martview). The motif analysis and transcription factor binding to promoter sequences were analyzed using the PLANTPAN3 (http://plantpan.itps.ncku.edu.tw/) database.

### Synteny and collinear analyses

Synteny analysis of protein family genes was performed between the *O. sativa* and *Arabidopsis thaliana* genomes, *O. sativa* and *H. vulgare*, and *O. sativa* and *Zea mays*. The gene-finding format (GFF) and genomic files of all the species were retrieved from Ensembl plants (https://ftp.ensemblgenomes.ebi.ac.uk/pub/plants/release-51), and the conserved regions of the genes present in the species genomes were identified using TBtools (Chen et al., 2020). The dual synteny analysis in TBtools or the one-step MCScanX tool was used to retrieve or present the evolutionarily conserved regions of the proteins in three species genomes. OsAHL duplications were found using the MCScanX (Multiple collinearity scan) software. For the protein sequences, BLAST was used with an e-value of 1.0e^−5^.

### *In silico* characterization and phylogenetic analysis

The phylogenetic relationship between rice AHL proteins was analyzed using neighbor-joining methods. Further, rice AHL proteins were compared with 29 *Arabidopsis* AHL proteins (Saitou and Nei, 1987). ClustalW in MEGA X software was used to align the AHL protein sequences (Kumar et al., 2016). A total of 1,000 bootstrapping values were used to obtain sequences (Felsenstein, 1985). The molecular weight, isoelectric point, subcellular localization, hydropathy index, and domains were predicted by the ExPasy assay (Gasteiger et al., 2005).

### Evolutionary patterns and divergence of the AHL gene family

Pairwise alignment of AHL gene encoding sequences allowed ClustalX 1.83 software to identify orthologous and paralogous sequences. TBtools was used to determine the number of synonymous and non-synonymous substitutions (Ks and Ka) at each site between the duplicate gene pairs. The duplication and divergence events of homologous genes and the selection pressure on duplicated genes were indicated, respectively, by the Ks and Ka values (Zhang et al., 2021). In order to estimate the dates of the gene-pair duplication events, the Ks rate was provided for the proxy time. Moreover, the times of the duplication events were calculated using the formula T = Ks/2λ, assuming that the clock-like rates of rice, represented by λ, were 6.65 × 10^−9^ substitutions/synonymous site/year.

### Tissue-specific expression study

The expression data were extracted from the RGAP database for *OsAHL* gene expression analysis, developmental stages, and stress conditions for transcriptome data. The data were normalized by log2-transformed mean data. These data were utilized for spatio-temporal expression of 19 genes of different tissues at distinctive stages of rice crop such as germination, seedling, tillering, booting, heading, blooming, and dough stage gene expression in response to drought and salt stress. Each gene data of Normalized Reads Per Kilobase Million (RPKM) values were retrieved. These values were transformed into log2 values. The RNA-seq data were critically analyzed, and heat maps were constructed using Genevestigator software (https://plants.genevestigator.com/local_plants/index.jsp).

### Digital, co-expression, and AHL protein interaction analysis

The protein–protein interaction (PPI) network of AHL proteins was elucidated and analyzed in the online interface of Search Tool for the Retrieval of Interacting Genes or Proteins (STRING) database version 10.5 using *O. sativa japonica* as the model plant (http://string-db.org/). The confidence score was set to a medium level of ≥0.4 for analysis. Further, digital expression profiling of OsAHL genes was performed from data obtained from the Rice Expression Profile (RiceXPro) v3 (http://ricexpro.dna.affrc.go.jp/) (Sato et al., 2013). Co-expression network analysis was performed using the RiceFREND database. The RiceFREND database was used for expression profiling of data obtained from microarray analysis of various tissues/organs under natural and stress conditions (Sato et al., 2013).

### Stress treatments, RNA extraction, and expression analysis using qRT-PCR

The two contrasting genotypes for heat IR64 (heat susceptible) and NL44 (heat tolerant) were chosen for the expression profile (Chaturvedi et al., 2017). The seeds of these genotypes were sown in a pot containing soil with six seeds in each pot. The pots were transferred to a growth chamber for germination (27°C ± 2°C). Fourteen-day-old germinated seedlings were exposed to drought and salt treatment in Murashige and Skoog liquid medium containing 20% PEG (6000) and 200 mM NaCl for a duration of 0 h (control), 2 h, and 4 h. All three biological replicates were maintained for each treatment (control, drought, and salt treatment). A total of 20 mg leaf samples was collected from each treatment with each replication for total RNA extraction. The RNA was extracted using a kit of HiMedia (HiPurA^®^Plant and Fungal RNA Miniprep Purification). The integrity of RNA was measured in agarose gel (1%) with formaldehyde. The purity and quality were estimated in the visible spectrophotometer at an absorption ratio OD260/OD280, and the reading value 1.8–2.0 indicated the presence of pure RNA in the tube (Kumari et al., 2023). RNA concentrations were estimated by NanoDrop (USA). The Moloney Murine Leukemia Virus Reverse Transcriptase (M-MuLV RT) enzymes were used to synthesize the complementary DNA (cDNA) first strand from the total RNA from both treated and control samples. The single-stranded cDNA was synthesized using the G-Biosciences Synthesis Kit (G-Biosciences Laboratories, St Louis, MO, USA). The optimization of PCR conditions was performed in the genomic DNA of rice using RT-PCR primers. The quantitative RT-PCR was performed using the cDNA of each treatment (control and drought- and salt-treated plants) as a template and using EvaGreen qPCR Master Mix (G-Biosciences Laboratories) in a quantitative real-time polymerase chain reaction. The qRT-PCR was performed using SYBR Premix Ex Taq II (Tli RNaseH Plus) (Takara Bio Inc., Mountain View, CA, USA) on the Mastercycler RealPlex system (Eppendorf) in three replicates. Rice 18S rRNA gene was used as an internal control and was used to normalize gene expression data of the *AHL* family of genes (Li et al., 2017). The qRT-PCR primer for all 20 genes of AHL was designed using the NCBI primer blast program (https://www.ncbi.nlm.nih.gov/tools/primer-blast/) (**Supplementary Table 1**). For each target gene, expression data were normalized with expression levels of 18S rRNA and calculated by formula 2^−ΔΔCt^ method. qRT-PCR data were obtained according to previous studies (Udvardi et al., 2008).

### Data analysis

Normalized data were used for one-way ANOVA to compare the treatments with the control in the IR64 genotype. The relative expression was calculated against the control for IR64 and NL44 genotypes and was compared by t-test. Graphs were made using MS Excel and GraphPad Prism version 8 (La Jolla, CA, USA) (Kumari et al., 2023).

## Results

### Genome-wide identification mapping, gene structure analysis, and *cis*-element analysis of *AHL* genes in rice

**Figure 1:**
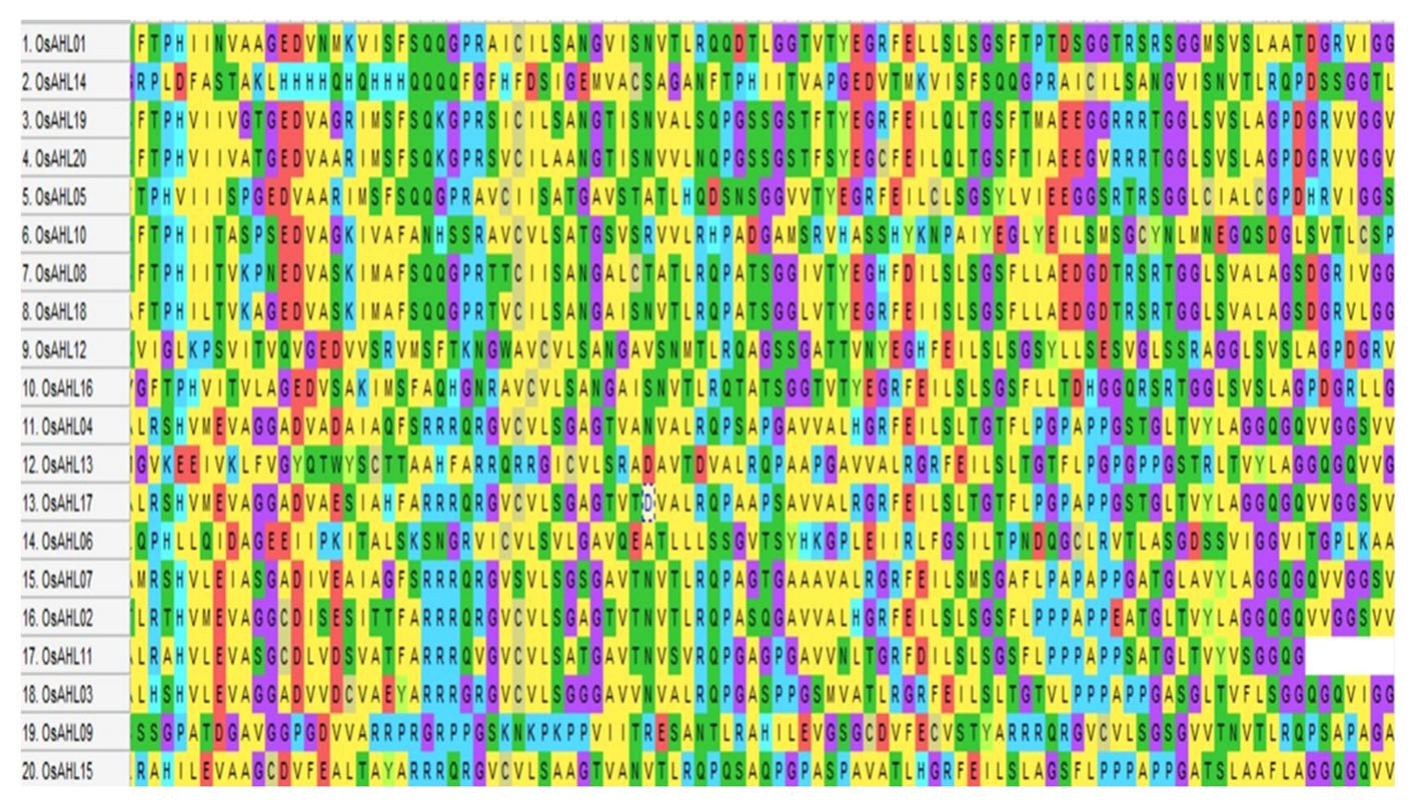
Representation of multiple sequence alignment of amino acid sequences of complete PPC domain for OsAHL transcription factors in rice.

**Figure 2:**
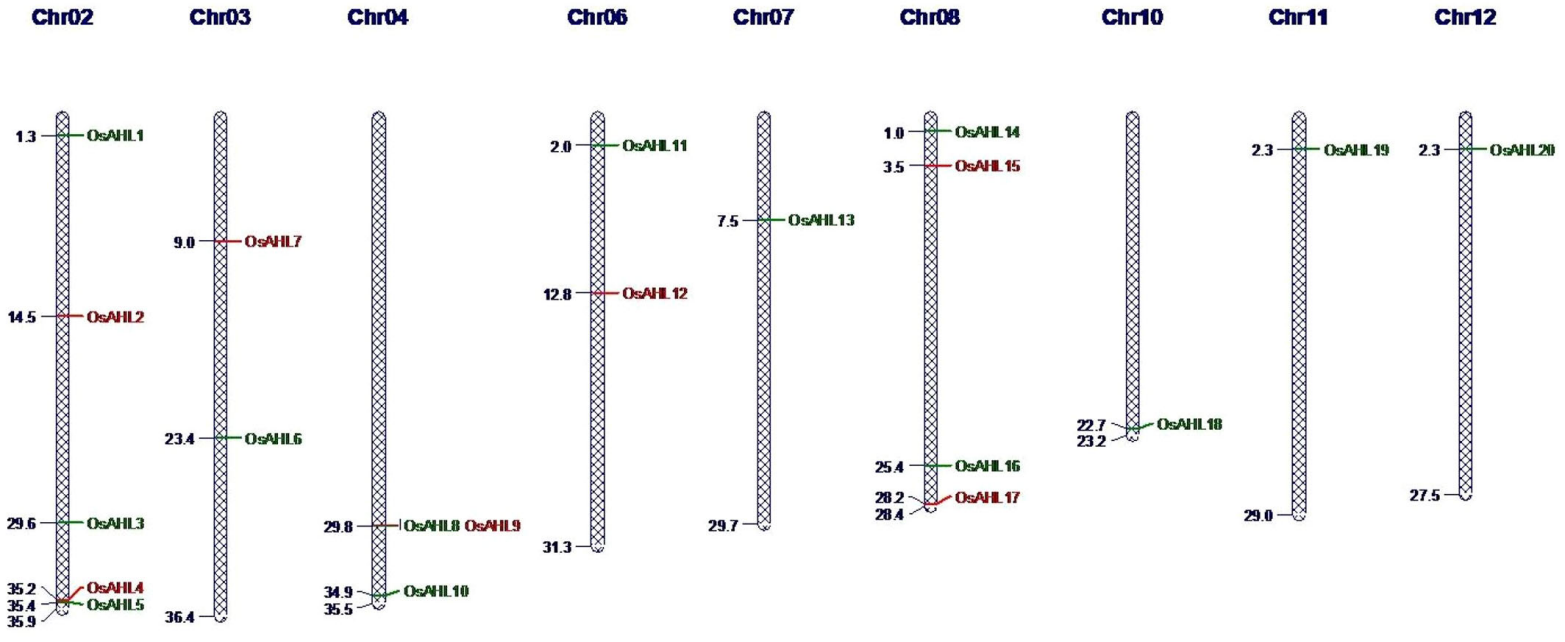
Distribution of *AHL* genes on rice chromosomes. Chromosome numbers are indicated on top.

In the present study, we conducted a comprehensive analysis of the *OsAHL* gene family in rice to determine their role in drought and salt stress responses as well as to understand the possible molecular function of *AHL*s in rice crops. We have identified 20 AHL protein sequences in rice that both contained PPC domain with AT-hook motif (RGAP database) (**Figure 1**). The distribution of all 20 genes of *OsAHL* was at only nine out of 12 chromosomes of rice. The result showed that chromosome 2 had a maximum of five genes followed by chromosome 8 with four genes, and chromosomes 3, 4, 6, and 10 had two genes. Chromosomes 7, 11, and 12 had only one gene (**Figure 2**).

**Figure 3:**
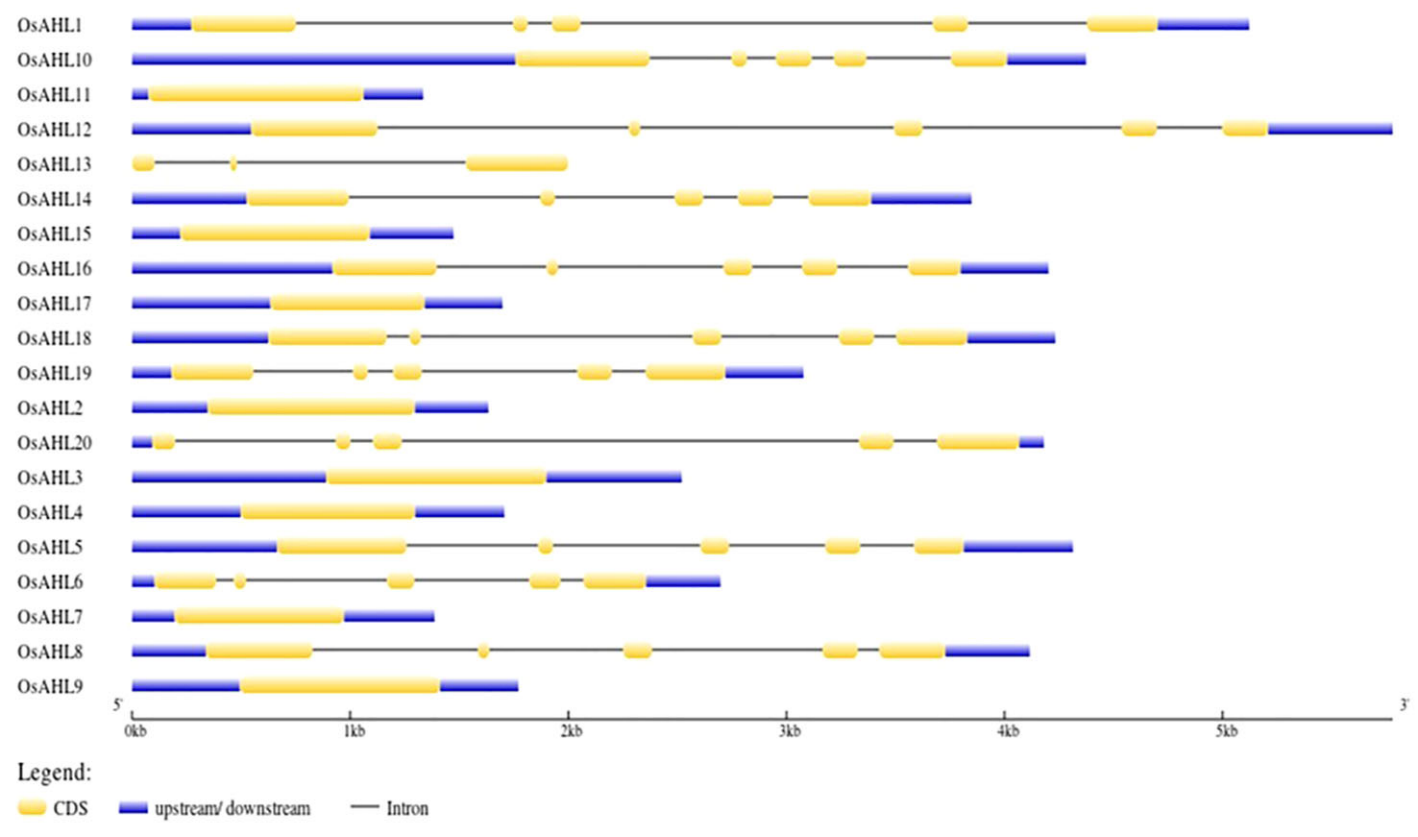
Gene structure analysis: intron/exon structure AHL gene from *Oryza sativa* L. Yellow color represents exon, and black line represents intron.

The gene structure analysis of Os*AHL* genes in rice showed different intron positions and lengths, and they were not conserved. The number of introns varied among Os*AHL* genes. Genes *OsAHL2*, *OsAHL4*, *OsAHL7*, *OsAHL9*, *OsAHL11*, *OsAHL15*, and *OsAHL17* did not contain any introns; genes *OsAHL1*, *OsAHL5*, *OsAHL6*, *OsAHL8*, *OsAHL10*, *OsAHL12*, *OsAHL14*, *OsAHL16*, *OsAHL18*, *OsAHL19*, and *OsAHL20* had the highest number of introns; *OsAHL13* had only two introns. In fact, *OsAHL1*, *OsAHL5*, *OsAHL6*, *OsAHL8*, *OsAHL10*, *OsAHL12*, *OsAHL14*, *OsAHL16*, *OsAHL18*, *OsAHL19*, and *OsAHL20* had the highest number of exons (**Figure 3**).

**Figure 4:**
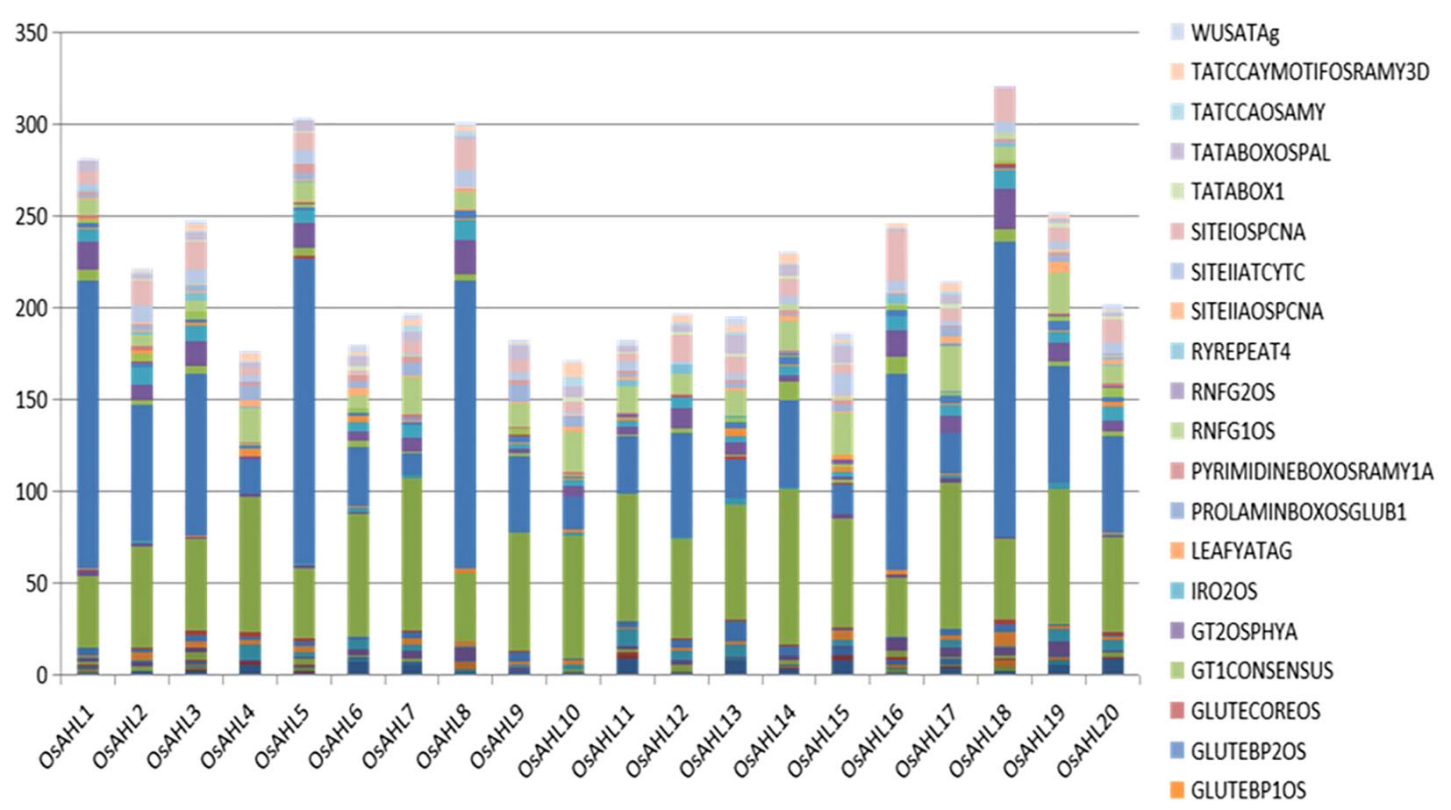
Representation of *cis*-motifs enriched in promoters of AHL gene homologs in rice. The X-axis represents the different genes of AHL family from the Rice Genome Annotation Project database. Y-axis denotes the number of motifs.

PlANTPAN3 was used to investigate the relationship between stress-related *cis*-elements and phytohormones in the *OsAHL* promoter region to better understand how *OsAHL* genes are regulated following abiotic stress and phytohormone treatments. There were 13 phytohormones and stress-related *cis*-elements found (**Figure 4**). In the *OsAHL* family, 17 of the 20 *OsAHL* genes included a *cis*-element associated with abiotic stress (LTR), as well as the MBS (drought-inducible element). *OsAHL3* and *OsAHL19* have two LTR *cis*-elements. Except for *OsAHL3* gene, all other OsAHL genes, i.e., *OsAHL3*, *6*, *8*, *9*, and *17*, are ABA-responsive genes that have ABA-responsive elements (ABRE). There are 10 ABREs in *OsAHL7* and other *OsAHL* genes with phytohormone-related responsive elements (**Supplementary Table 2**).

### Phylogenetic analysis revealed diversification of AHL proteins

**Figure 5:**
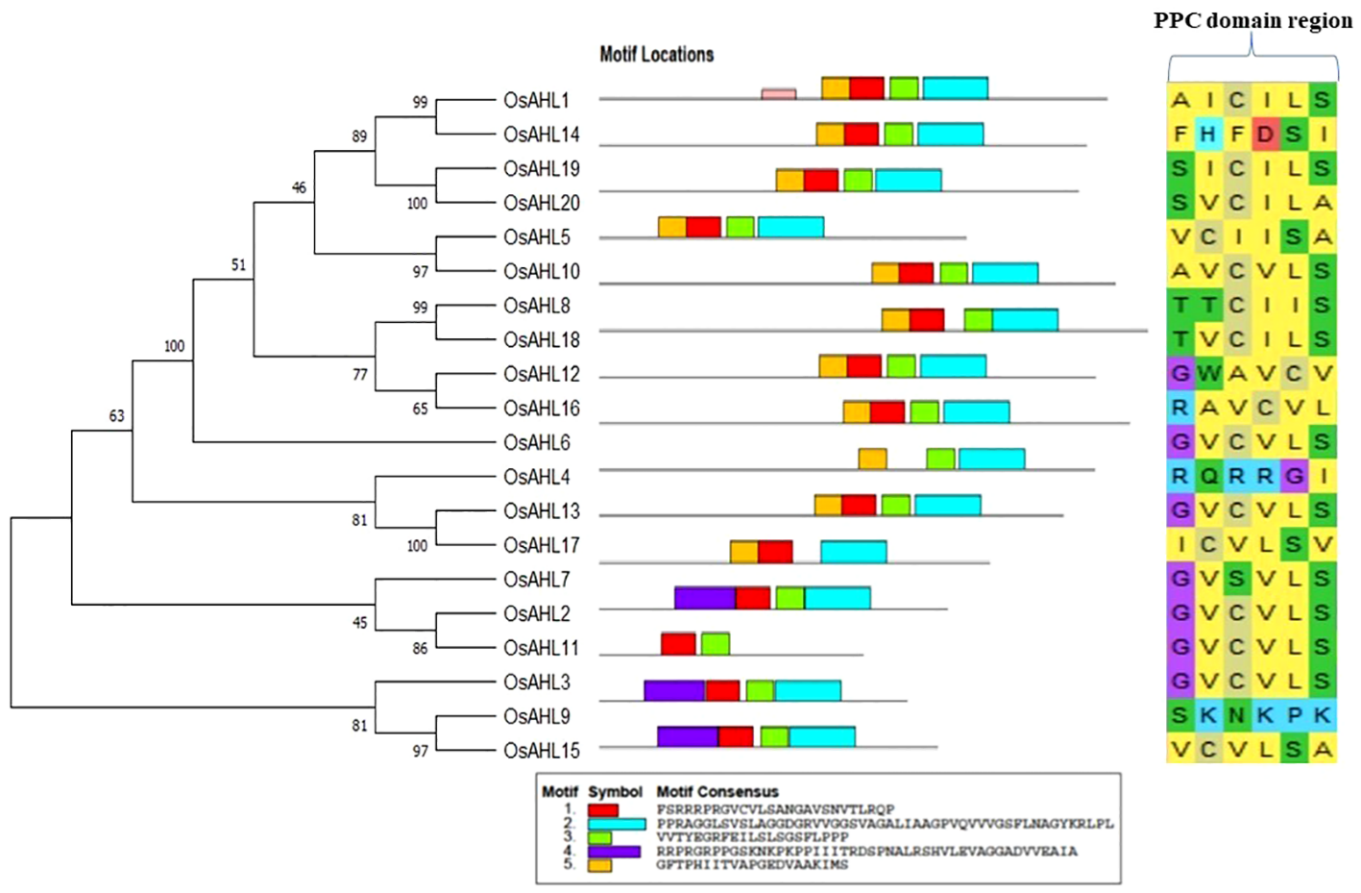
Phylogenetic relationships between AHL family of transcription factors (TFs) in rice. The bootstrap test was performed with 1,000 iterations and presented on the node. Phylogenetic tree is shown on the left; characteristic sequences in two of the functional units of the AHL proteins, AT-hook motif and most conserved PPC domain, are shown on the right.

**Figure 6:**
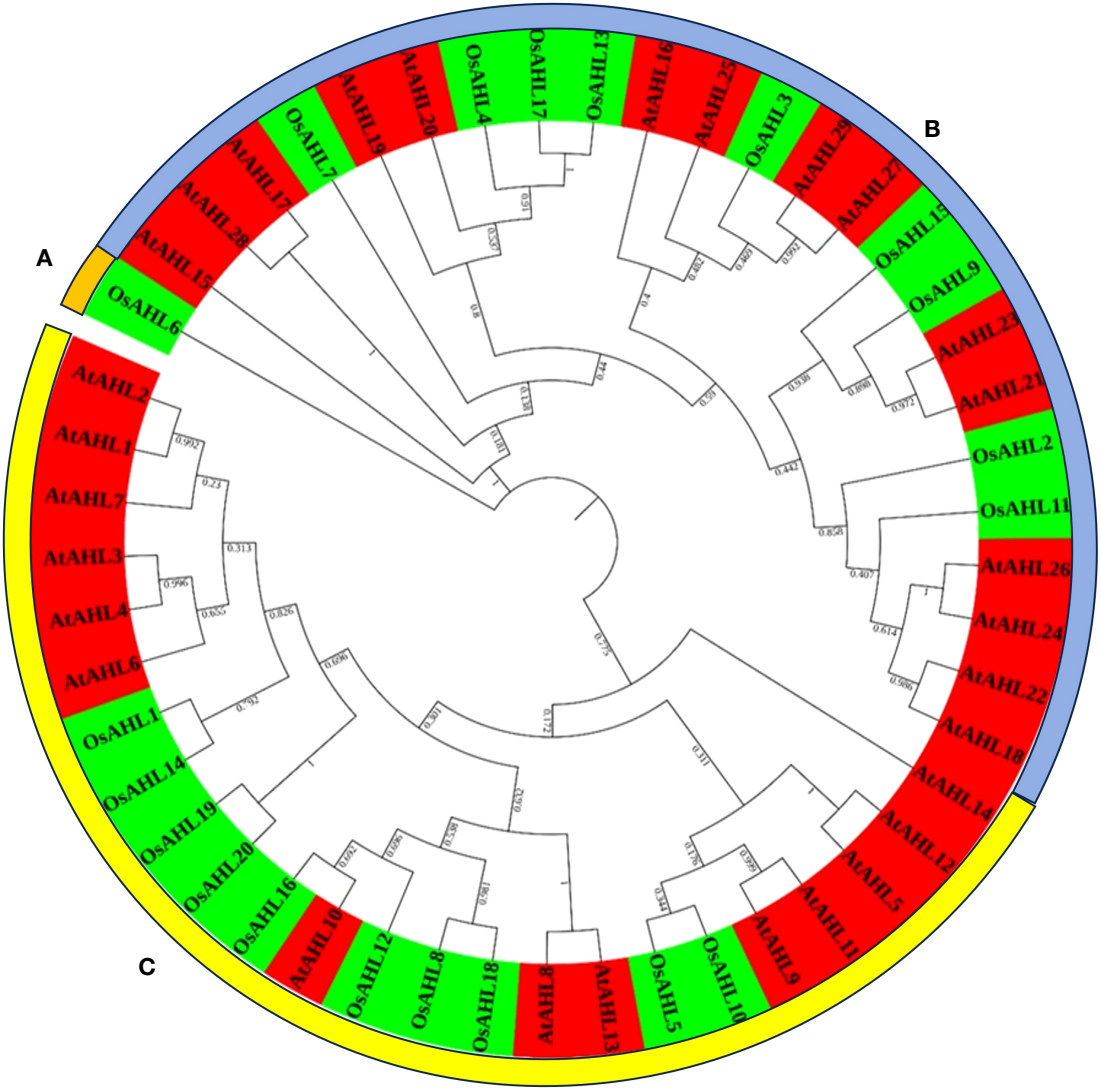
Phylogenetic tree of the AHL gene family across *Arabidopsis* and rice. The unrooted phylogenetic tree was constructed using raxMLGUI 2.0 and the maximum likelihood method. The bootstrap test was performed with 1,000 iterations and presented on the node. Two different colors are used to represent two different species.

The evolutionary relationship between the AHL proteins of rice was determined. It was classified based on the presence of characteristic motif sequences. The phylogenetic analysis was performed using the full-length sequences of protein. The examination of multiple sequence alignment presence or combination of the PPC domain, and the AT-hook motif functional unit result revealed no clear clade differentiation of PPC domains of AHL proteins in rice (**Figure 5**). The rice AHL family of transcription factors was also compared with *Arabidopsis*. It represents the relation of the AHL transcription factors between the dicot (*Arabidopsis*) and monocot (rice) crops (**Figure 6**). The phylogenetic tree of the AHL transcription factor was divided into three clades. Clade A contained only one transcription factor (OsAHL6), clade B contained nine transcription factors, and clade C contained 10 transcription factors of rice. The physical and chemical properties of the AHL family of transcription factors of rice were analyzed, and the number of amino acids of OsAHL protein in rice varies from 201 to 420 amino acids. The molecular weight of 20 OsAHL proteins varies from 20,510.56 kDa to 42,840.10 kDa. Out of these, OsAHL10 (420 amino acids) had the highest number of amino acids with a molecular weight of 42,840.10 kDa, and OsAHL13 had the lowest number (201 amino acids) of amino acids with a molecular weight of 20,510.56 kDa. The theoretical pI values for the *AHL* gene family were generally less than 10%, except for OsAHL12 (10.46) and OsAHL16 (10.08). Hence, in *AHL* gene family, all of the proteins are unstable. Proteins having an instability index of less than 40 are considered stable. The grand average of the hydropathy index showed a negative value, indicating that all the AHL transcription factors are non-polar in nature (**Supplementary Table 3**). The gene sequence length varies from 1,335 bp to 5,779 bp, and OsAHL12 (1,335 bp) had the largest gene length, while *OsAHL11* (1,335 bp) showed the lowest gene length. Plant-mPLoc studies were conducted for the cellular localization of AHL proteins. The result showed that most of the AHL proteins in rice were restricted to nuclei in subcellular localization. Out of 20 *OsAHL*, 13 genes were solely found in nuclei, *OsAHL5* was found in the cytoplasm, and *OsAHL13* and *OsAHL15* genes were found in the chloroplast. A total of four AHL genes (*OsAHL2*, *OsAHL3*, *OsAHL10*, and *OsAHL17*) were found in both the chloroplast and nucleus (**Supplementary Table 4**).

### Gene expression patterns provide key insight into AHL gene functions

**Figure 7:**
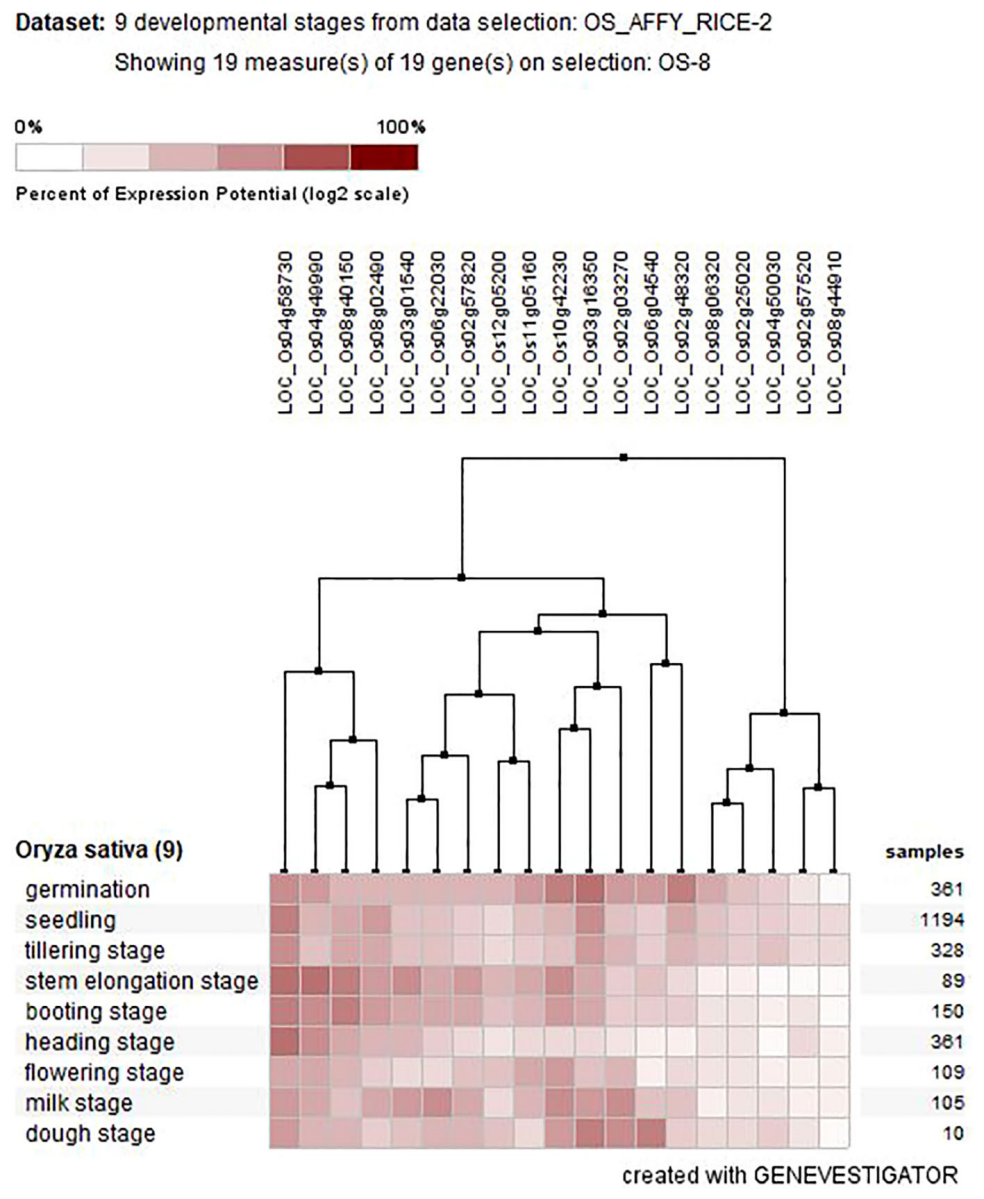
Tissue-specific expression of AHL genes at different developmental stages of *Oryza sativa* L.

To obtain clues about the role of *OsAHL* genes in rice development, we studied temporal and spatial expression patterns of the Os*AHLs* in 19 distinct transcriptomes in rice. Out of 20 *OsAHL* genes, 19 were expressed in nine developmental stages (vegetative and reproductive tissues). The result suggested the diverse roles of Os*AHL* genes in the vegetative and reproductive phases of plant development. The number of genes expressed differently at different developmental stages such as germination (three genes), seedling tillering (two genes), stem elongation stage (five genes), booting stage (three genes), heading stage (two genes), flowering stage (one gene), milk stage (seven genes), and dough stage (five genes) (**Figure 7**).

### Synteny and collinear analyses

**Figure 8:**
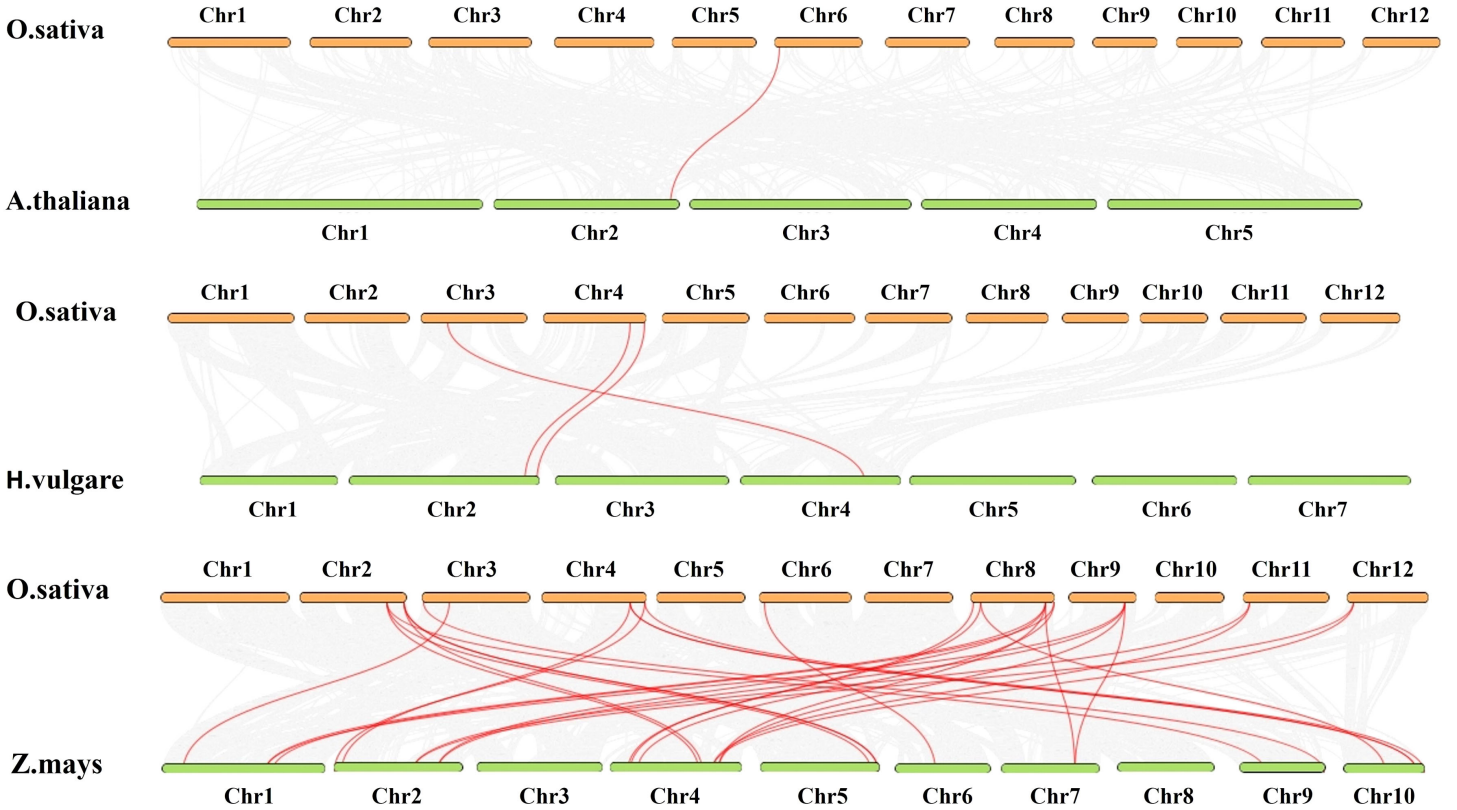
Gene duplication and synteny analysis of AHL genes between *Oryza sativa* and *Arabidopsis thaliana*, *Hordeum vulgare*, and *Zea mays*. The figure depicts the gene duplication and synteny analysis between AHL genes in *O. sativa* and *A. thaliana*, *H. vulgare*, and *Z. mays* genomes. In the background, gray lines represent collinear blocks within genomes, indicating regions of conserved gene order and orientation. These collinear blocks suggest evolutionary relationships and shared ancestry between species. The red lines highlight the syntenic AHL gene pairs, indicating the presence of orthologous AHL genes between *O. sativa* and *A. thaliana*, *H. vulgare*, and *Z. mays* genomes. These syntenic AHL gene pairs exhibit conserved gene positions and likely share functional similarities. The gene duplication events within the AHL gene family are demonstrated by the presence of duplicated gene pairs within species. These gene duplication events contribute to the expansion and diversification of the AHL gene family in *O. sativa* and *A. thaliana*, *H. vulgare*, and *Z. mays* genomes. The gene duplication and synteny analysis provide valuable insights into the evolutionary relationships, shared ancestry, and conservation of AHL genes between *O. sativa* and *A. thaliana*, *H. vulgare*, and *Z. mays* genomes, shedding light on the functional conservation and divergence of AHL genes across these plant species.

The BLASTP and MCScanX approaches were used to identify the collinearity between the rice *AHL* gene family and to identify a likely relationship between the rice *AHL* genes and a potential duplication event. In order to investigate the potential evolutionary routes of the rice *AHL* gene family, comparative synteny maps were generated for *O. sativa* related to *A. thaliana*, *Zea mays*, and *H. vulgare* (**Figure 8**). Finally, one collinear gene pair of AHL was found between *Arabidopsis* and rice (*OsAHL11*), 18 collinear gene pairs were found between rice and *Z. mays* (*OsAHL1*, *OsAHL2*, *OsAHL3*, *OsAHL4*, *OsAHL5*, *OsAHL6*, *OsAHL7*, *OsAHL8*, *OsAHL9*, *OsAHL10*, *OsAHL11*, *OsAHL14*, *OsAHL15*, *OsAHL16*, *OsAHL17*, *OsAHL18*, *OsAHL19*, and *OsAHL20*), and three collinear gene pairs were found between rice and *H. vulgare* (*OsAHL7*, *OsAHL8*, and *OsAHL10*) (**Figure 8**). The synteny conservation between the rice and *Z. mays* genomes is the highest; this finding may indicate that *OsAHL* and *ZmAHL* genes in rice have similar structures and functions.

### Gene ontology annotation, duplication, and divergence rate, and co-expression and protein network analyses of AHL genes

**Figure 9:**
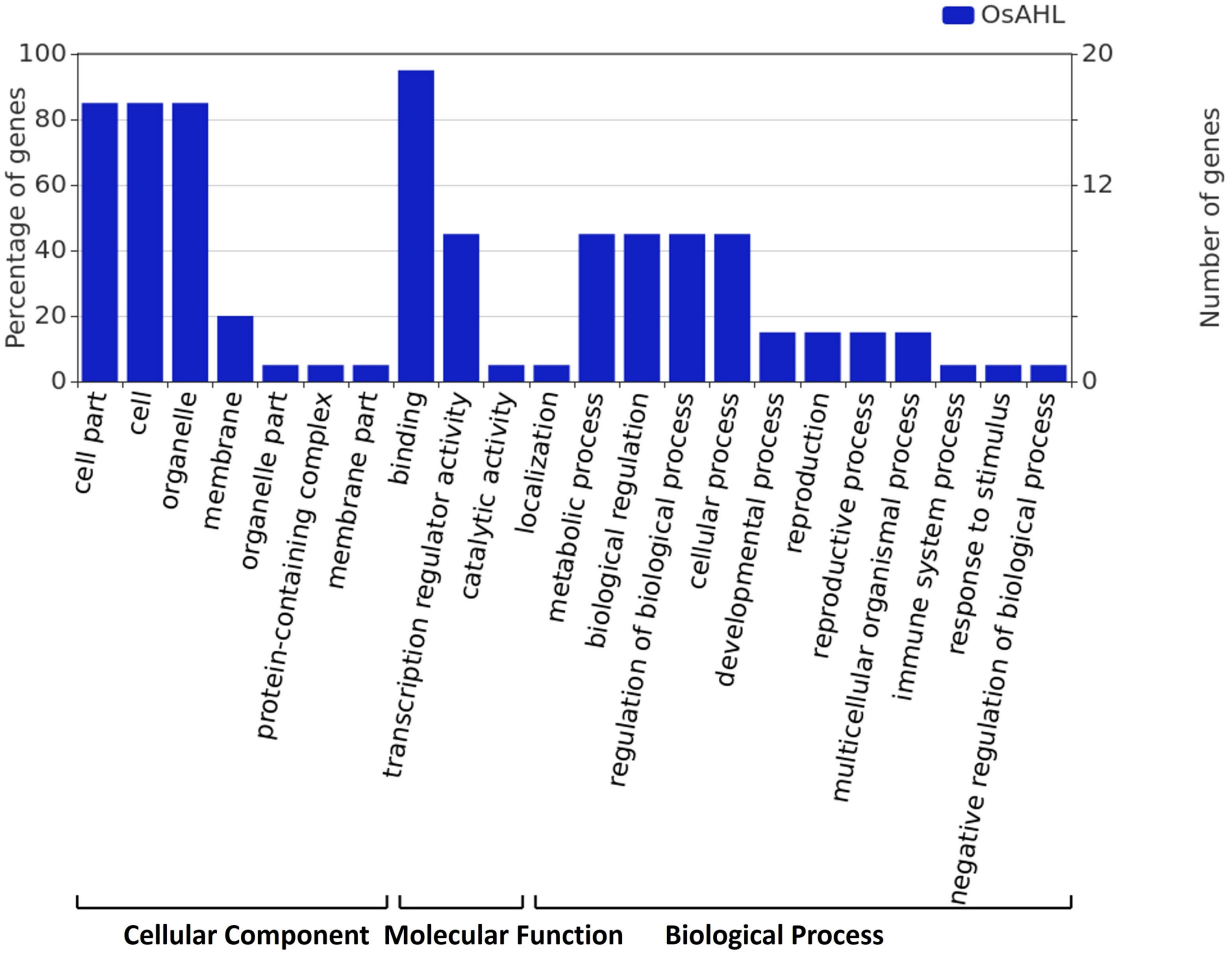
Gene ontology annotation of AHL proteins. The Blast2GO output defines the cellular components, molecular function, and biological process of 20 AHL proteins.

The gene ontology (GO) analysis was performed for rice OsAHL protein in diverse biological, cellular, and molecular functions. GO analysis showed that *OsAHL* genes are crucial for a range of biological processes. The majority of OsAHL genes are found in cellular followed by biological processes. The analysis of biological processes mediated by AHLs depicted that most AHL proteins were involved in the regulation of DNA template transcription and the development stage of rice crops. Further, the cellular component analysis revealed the localization of AHL proteins in the nucleus, membrane, and signal recognition particle receptor complex (**Figure 9**).

The TBtools were used to calculate duplication events of AHL gene pairs in rice. Only segmental duplication events were reported between AHL of rice. The association of Darwinian positive selection in duplication and divergence of AHL in rice was explored by estimating the ratios of non-synonymous (Ka) versus synonymous (Ks) substitution rate (Ka/Ks) of eight gene pairs of rice *AHL* gene. The Ka/Ks ratio for segmentally duplicated gene pairs in rice AHL genes ranged from 0.17 to 0.45 with an average value of 0.30. Further, the duplication event of these AHL segmentally duplicated genes may be estimated to have occurred approximately 54 MYA (**Table 1**).

**Table 1:** Time of duplication and divergence (MYA) based on synonymous substitution rate (Ka) and nonsynonymous substitution rate (Ks) estimated using paralogous and orthologous OsAHL gene pairs.

OsAHL group	Chromosomal location	Duplication type	Ka	Ks	Ka/Ks	Time (MYA)
OsAHL1–OsAHL14	Chr 2 Chr 8	Segmental	0.27	0.97	0.28	72.97
OsAHL19–OsAHL20	Chr 4 Chr 12	Segmental	0.13	0.50	0.26	37.52
OsAHL5–OsAHL10	Chr 2 Chr 10	Segmental	0.46	2.31	0.20	173.89
OsAHL8–OsAHL18	Chr 4 Chr 10	Segmental	0.32	1.82	0.17	137.19
OsAHL16–OsAHL12	Chr 8 Chr 6	Segmental	0.42	0.94	0.45	70.61
OsAHL2–OsAHL11	Chr 2 Chr 6	Segmental	0.34	0.97	0.35	73.10
OsAHL9–OsAHL15	Chr 4 Chr 8	Segmental	0.21	0.86	0.24	64.56
OsAHL17–OsAHL13	Chr 8 Chr 7	Segmental	0.08	0.18	0.44	13.82

T = Ks/2x, where x is 6.65 × 10^−9^.

MYA, million years ago.

**Figure 10:**
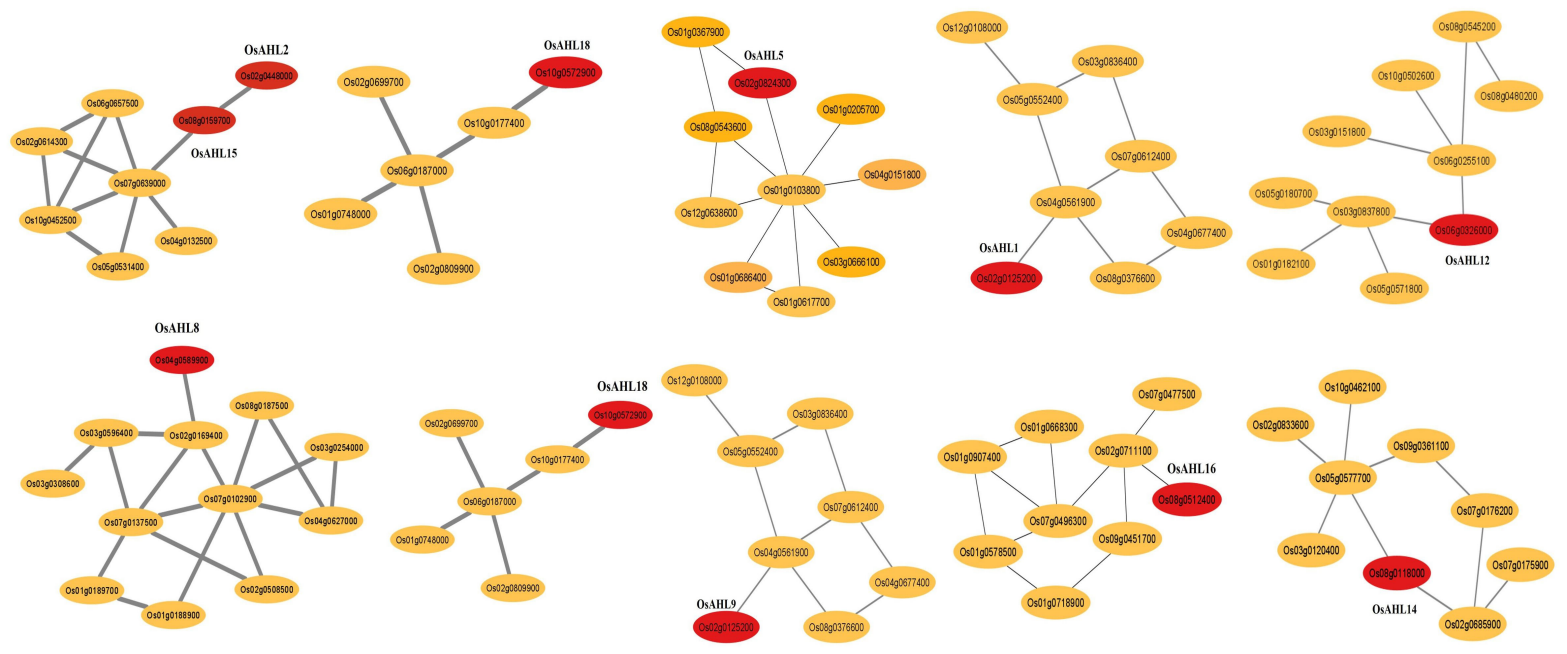
Co-expression networks of OsAHL genes (red circles). Each network was generated using the data on RiceFREND gene co-expression database.

To determine whether the genes are either regulated by *ALH*s or co-regulators to modulate the expression of downstream genes, we conducted a co-expression analysis. *AHL* genes were co-expressed with 12 other genes that play different developmental and abiotic stress tolerance. For example, *Os07g0639000* (peroxidase activity) was co-expressed with *OsAHL15* gene, and peroxidase comes under the oxidoreductase family and is an antioxidant enzyme. It is induced by wounding and ethylene treatments (de Obeso et al., 2003). *Os10g0177400* (HVA22-like protein) co-expressed with *OsAHL18* gene and its function in iron homeostasis (Sudre et al., 2013). *OsAHL14* co-expressed with *Os05g0577700* (serine/threonine protein kinase) and *Os02g0685900* (calcium-dependent protein kinase) genes. The Ser/Thr kinase and calcium-dependent protein kinase family is one of the most important kinase families and is found in all eukaryotic genomes. It plays a role in the modulation of kinase activity by external stimuli and stress conditions (Asano et al., 2005; Hwang et al., 2018). *OsAHL5* gene was co-expressed with Os01g0367900 (chromatin remodeling complex) and Os01g0103800 (uncharacterized proteins). *Os01g0367900* gene possesses intrinsic activity of ATP-dependent nucleosome remodeling. It possesses a catalytic subunit of several complexes that are able to form *in vitro* nucleosome arrays on chromatin (Huang et al., 2018). *Os04g0561900* gene co-expressed with *OsAHL1* and its code for l-ascorbate oxidase and acts as an antioxidant (**Figure 10**).

**Figure 11:**
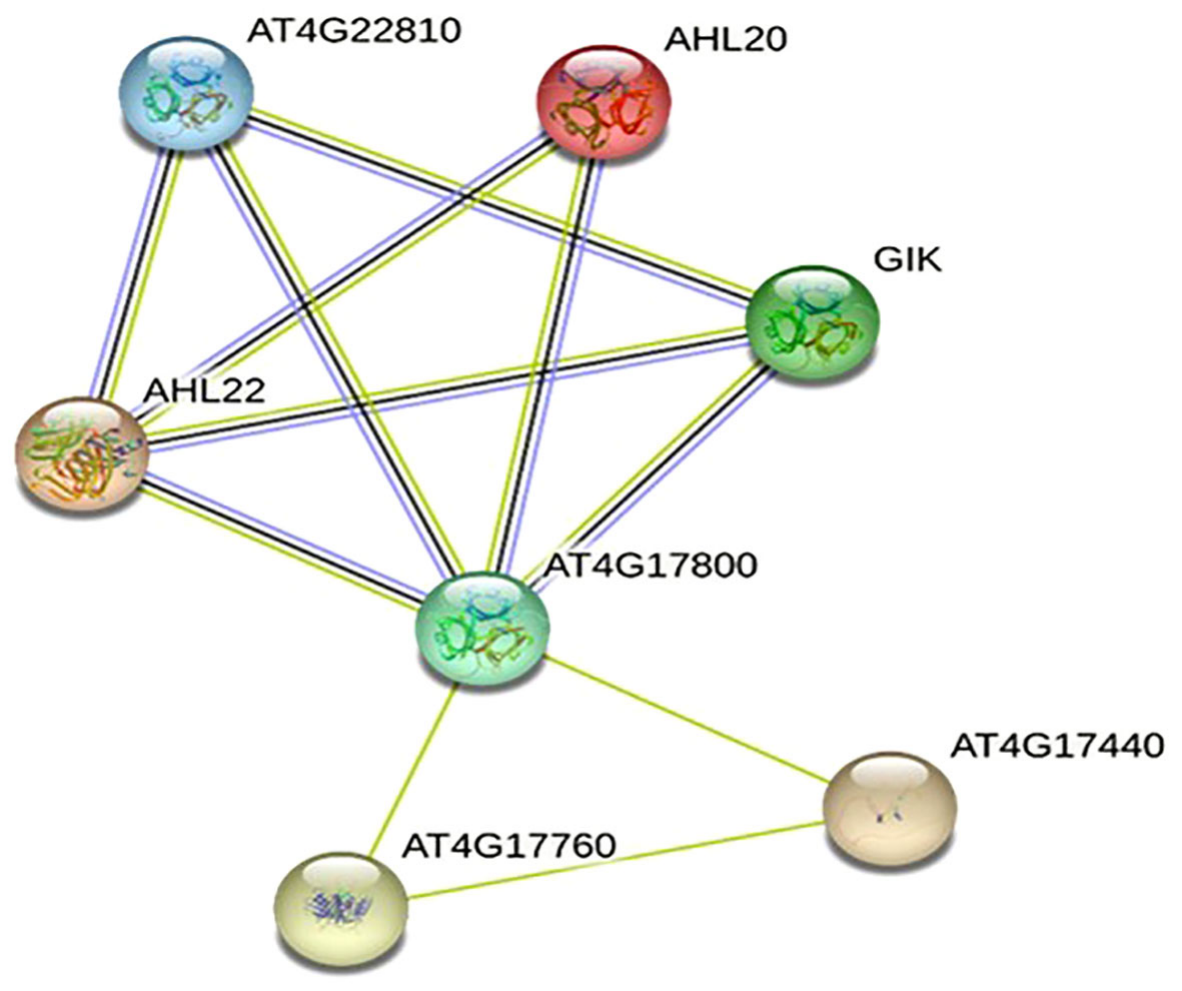
Predicted protein–protein interaction network of OsAHL proteins in rice. Edges between nodes represent protein–protein associations predicted by experimentally determining (pink line) from curated databases (blue line) and text mining (green and dark blue) gene co-occurrence. Table represents gene ontology. AT4G22810: putative AT-hook DNA-binding family protein. AHL20: overexpression results in early flowering in short and long days. GIK: direct target of AGAMOUS; regulates patterning and differentiation of reproductive organs. AHL22: overexpression of the gene results in delayed flowering; it is involved in both photo- and skotomorphogenesis. AT4G17800: putative AT-hook DNA-binding family protein. AT4G17760: PCNA domain-containing protein. AT4G17440: cleaves the C-terminal extension of the D1 precursor (pD1) to form mature D1; initiation of the formation of the oxygenic D1/D2 type PSII.

A PPI network was created using the STRING database to investigate the relationships among the 20 protein sequences of OsAHLs. After filtration, seven unique interactions of OsAHL proteins with diverse proteins such as AT4G22810 (AHL24), AHL20, GAINT KILLER (GIK), AHL22, AT4G17800 (AHL23), AT4G17760, and AT4G17440 are depicted in **Figure 11**. The protein AT4G17800 exhibits the highest number of nodes and is shared in its interactions with most other proteins. Further, the PPI network also depicts the activation of stress-related proteins including AHL20, AT4G17800 (AHL23), AT4G22810 (AHL24), and GIK. Further, the interaction of different OsAHL proteins with developmental proteins regulates processes such as flowering and differentiation of reproductive organs.

### Expression profiling of AHL genes in contrasting genotypes of rice

The expression profiling of 20 Os*AHLs* in response to drought and salt stress was determined using quantitative real-time PCR analysis. The expression of all the genes was normalized with reference to the expression of the 18 S rRNA gene. Quantitative real-time PCR-based expression analysis of *AHL* genes was conducted in the IR64 genotype of rice under drought and salinity stresses, and the expression pattern was compared with the control. Genes *OsAHL1*, *OsAHL17*, and *OsAHL18* showed significant upregulation after 4 h of drought treatment and 2 h and 4 h of salinity treatment as compared to the control. Drought for 4 h and salinity for 4 h of treatment of *OsAHL4*, *OsAHL16*, *OsAHL13*, *OsAHL16*, *OsAHL19*, and *OsAHL20* genes showed significant upregulation as compared to the control in the IR64 genotype. However, *OsAHL2*, *OsAHL8*, *OsAHL10*, and *OsAHL12* showed significant upregulation after 2 h of drought treatments, while *OsAHL9* and *OsAHL15* showed upregulation after 4 h of drought treatment as compared to the control in the IR64 genotype. In contrast, genes *OsAHL1* and *OsAHL14* showed significant downregulation after 2 h of drought treatments. Gene *OsAHL3* showed significant upregulation after 2 h of drought and 2 h and 4 h of salinity treatments over the control. *OsAHL5* showed significant upregulation after 2 h and 4 h of drought treatment compared to the control in IR64. Similarly, gene *OsAHL7* showed significant upregulation after 2 h and 4 h of drought and 4 h of salinity treatments. *OsAHL11* and *OsAHL14* genes showed significant upregulation after 2 h and 4 h of treatments of salt over the control (**Supplementary Figure 1**). The result clearly showed that all 20 *OsAHL* genes upregulated after either drought or salt or both treatments.

**Figure 12:**
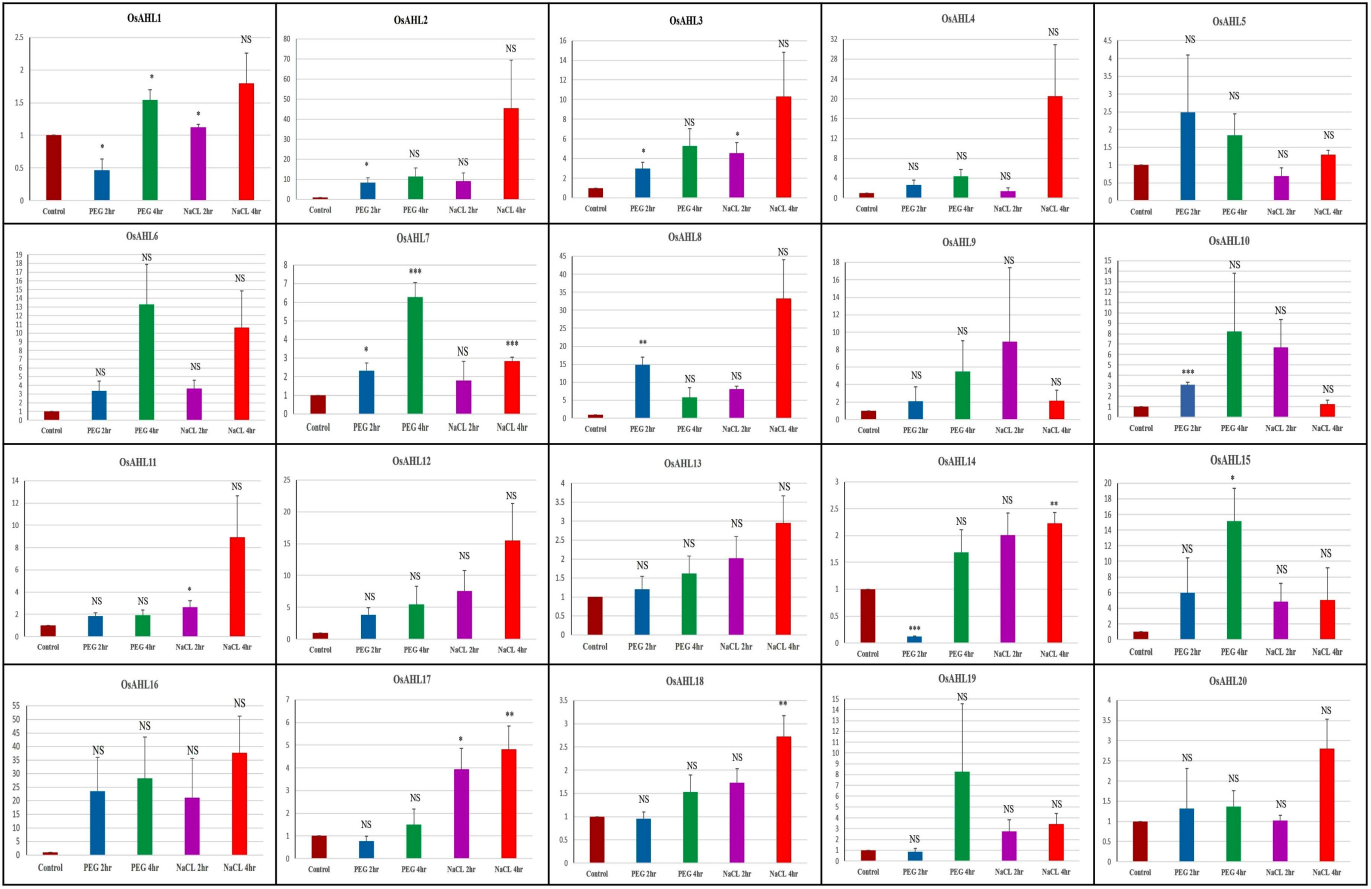
Differential gene expression of 20 OsAHL family genes under drought and salinity stress in two rice genotypes, IR64 and NL44. Y-axis represents the log2 fold relative expression level. The names of the genes are given at the bottom. The data are the average of three technical replicates. The error bar indicates the standard deviation between three technical replicates. Statistical comparison between two lines is shown in the graph, where *p < 0.05 and **p < 0.01 are the significant differences, and NS means non-significant differences.

The differential Os*AHL* gene expression in IR64 and NL44 was compared using a t-test under drought and salt stress conditions. A total eight genes (*OsAHL1*, *OsAHL2*, *OsAHL3*, *OsAHL10*, *OsAHL14*, *OsAHL17*, *OsAHL18*, and *OsAHL19*) showed significant upregulation in NL44 compared to IR64 after 2 h of drought treatment. Similarly, a total of seven genes (*OsAHL7*, *OsAHL10*, *OsAHL13*, *OsAHL14*, *OsAHL17*, *OsAHL18*, and *OsAHL20*) showed significant upregulation in NL44 compared to IR64 after 4 h of drought treatment. The differential expression pattern under salt stress was also compared for the AHL group of genes in NL44 and IR64 genotypes (**Figure 12**). A total of three genes (*OsAHL10*, *OsAHL13*, and *OsAHL20*) showed significant upregulation after 4 h of salinity treatment in genotype NL44 as compared to IR64. Interestingly, *OsAHL8* gene was significantly downregulated after 2 h of drought treatment in NL44 as compared to IR64. However, *OsAHL14* gene was significantly downregulated after 2 h and 4 h of salinity treatment in NL44 as compared to IR64 (**Figure 12**).

## Discussion

In recent years, due to advancements in sequencing technologies, the cost of sequencing genomes reduced, leading to the frequent addition of sequence information in repositories along with a number of unannotated hypothetical genes (Punta et al., 2012). It is very important to understand the function of these types of hypothetical proteins. It will enhance our knowledge of biological systems. In order to understand the gene structure and function of these unknown genes, their family analysis is a very effective strategy. In the present study, a total of 20 *AHL* genes were identified in *O. sativa*, as similarly reported by Kim et al. (2011), and a total of 29 *AHL* in *A. thaliana* (Zhao et al., 2014), 37 in *Populus trichocarpa* (Zhao J., 2013; Wang et al., 2021), 48 in *Gossypium raimondii*, 51 *Gossypium arboretum*, and 99 in *Gossypium hirsutum* (Zhao et al., 2020). The number of AHL genes identified different numbers in rice (Zhao et al., 2014; Bishop et al., 2020). Recently, a total number of 26 Os*AHL* genes were identified in rice (Kumar et al., 2023). The number of *AHL* genes varied in different crops even within the crop. The PPC domain of *AHL* genes showed a divergence nature of domain in the rest of the *AHL*s. Thus, it demonstrated the expansion of *AHL*s’ biological roles. The majority of AHLs were found to be located in the nucleus, which is consistent with these proteins binding to an AT-rich region of DNA (Lim et al., 2007). Phylogenetic analysis was used to establish the evolutionary connection among OsAHL proteins. The degree of AHL divergence in rice compared to other plants was well demonstrated by the data. The *Arabidopsis* genome has 29 *AHL* genes in all that we have annotated. Previous identified similar number of *AHL* genes (Matsushita et al., 2007). Analysis of rice and *Arabidopsis* protein sequences and phylogenetic relationships revealed that the AHL proteins exhibit both sequential and phylogenetic divergences (Kawashima et al., 2005). Because genes with comparable structures may have developed from a single ancestor, a gene’s capacity to code defines its structure and provides information about gene ancestry. The detected genes’ exon–intron patterns were revealed by the gene structure analysis. Intron distribution and number varied among subfamilies. Genes belonging to the *AHL* family typically had more enduring patterns and simpler structures. The *AHL* family in rice exhibits structural variation, according to a structural investigation of *OsAHL* genes. Genes’ introns are a crucial and significant structural element. It plays important functions in alternative splicing and intron-mediated enhancer-mediated regulation of gene expression. It makes natural selection more effective (Deshmukh et al., 2016). It has been observed that intron-deficient genes, which most likely result from retro-transposition, typically arise by a copy–paste method (Deshmukh et al., 2016).

The promoter area of important stress-responsive transcription factors was searched for *cis*-elements, which are known to regulate stress-responsive genes in order to gain insight into the regulation of AHL genes. All OsAHL family members have at least a phytohormone-linked or stress-linked *cis*-element, specifying their function in response regulation. LTR and MBS *cis*-elements were also discovered in a number of OsAHL promoters, including OsAHL3, 5, 6, 9, 14, and 19, and OsAHL2, 3, 7, 8, 9,12, 13, 16, 18, and 19, respectively. Numerous significant hormone-regulated, defense-responsive *cis*-elements were identified by motif analysis. Numerous genes with biotic and abiotic stress-responsive *cis*-motifs were discovered to be concentrated in the *AHL* gene promoters. They control a multitude of stress tolerance signaling pathways that upregulate the stress response of *OsAHL* gene. According to the studies, AHL controls the growth and development of plants and also plays a role in NCR gene expression and nodule development in *Medicago* (Zhao et al., 2013; Zhang et al., 2023).

Tandem and segmental duplications of the whole genome generally provide multiple copies of genes in a given family of genes. Such types of gene duplication events are reported in different TF families including AP2/ERF, MYB, and NAC (Cannon et al., 2004; Licausi et al., 2010; Puranik et al., 2013; Yadav et al., 2014; Mishra et al., 2014). The Ka and Ks values measured the divergence after duplication. The ratio of Ka/Ks value measures the selection pressure subjected to gene pairs. A ratio of Ka/Ks of less than one means purifying selection, a value more than one means accelerated evolution, and Ka/Ks = 1 indicates neutral selection (Lynch and Conery, 2000). Ka/Ks ratios were estimated to be smaller than one in the current investigation. It suggests that there was significant purifying selection pressure acting on the redundant *AHL* genes. During the process of evolution, it underwent substitution elimination and severe selective constraints from natural selection. Overall, it can be said that the evolution of big gene families, such as *AHL* genes in rice, is limited to segmental duplication events (Cannon et al., 2004).

Co-expression analysis showed that *OsAHL* genes co-expressed with reactive oxygen species (ROS) scavengers (peroxidase), a signaling molecule (Ser/Thr kinase and calcium-dependent protein kinase) related to stress tolerance, and other genes are functionally uncharacterized. All of those genes are not functionally characterized and are used as novel candidate genes. Previous studies showed that *AHL* gene also plays a role in growth, development, and abiotic stress tolerance (Wang et al., 2021; Zhang et al., 2023). Abiotic stress (drought and salinity) is controlled by multiple genes (Atkison et al., 2013). Since TFs respond to abiotic stress very quickly, many downstream genes were involved in the abiotic stress tolerance expression. The protein network analysis of OsAHLs suggested that they interact with different proteins that perform diverse functions such as flowering regulation, reproductive organ development, and photosynthesis activity. OsAHLs showed consistency with the predicted interactions through their PPC domain. Zhao et al. (2020) reported elevated expression of GIANT KILLER (GIK), responsible for encoding an AHL protein that led to significant reproductive abnormalities and the suppression of genes related to the formation and specialization of reproductive flower parts. However, a study conducted by Kumar et al. (2023) showed expression of OsAHL20 under salinity conditions and reported enhanced expression of AT4G17800 (AHL23) and AT4G22810 (AHL24) in leaves and roots, indicating their potential involvement in the plant’s response to drought stress. Another study suggested the overexpression of *Arabidopsis* ATHG1/AHL23 and ATPG3/AHL20 genes in association with salt stress tolerance in *Zostera japonica* (Jeong et al., 2020). However, few predicted interactions involving proteins other than AHL are novel and need functional characterizations to reveal their role in rice development (Zhao et al., 2013).

The present investigations also explore the roles of various genes in specific tissue, and they will significantly improve our knowledge of both the evolution and functional aspects of *AHL* genes. The annotation of rice AHLs is supported by the transcriptome data set of all the expressed genes identified in our study. Differential expression patterns of *OsAHL* genes by hormones and stresses suggest that they play different roles during biotic and abiotic stress conditions and in the development of plants.

*AHL* genes play important roles in plant development, floral transition, and response to stresses (Jin et al., 2011; Zhao et al., 2013; Zhao et al., 2014). The functional validation was conducted in heat-contrasting genotypes of rice such as IR64 and NL44 (Chaturvedi et al., 2018). The gene expression in IR64 showed that all genes (100%) are significantly upregulated under drought stress, and 80% of genes are significantly upregulated under salt stress over the control. The result clearly showed the role of *OsAHL* genes under drought and salt stress. *OsAHL1* gene enhances drought resistance in rice by regulating drought-related genes during the panicle stage of development (Zhao et al., 2016). These results are also consistent with Wang et al. (2021), who reported the role of *AHL* genes in drought tolerance. Further, differential expression of *AHL* genes in NL44 and IR64 confirmed the contrasting nature of drought and salt tolerance of these genotypes and suggested that NL44 is highly tolerant of drought and salt stress.

## Conclusion

In the present study, a total of 20 *OsAHL* genes were identified. AHLs can be better understood by looking at the phylogeny, conserved characteristics, and protein interaction network in-depth. Furthermore, a methodical approach was used to investigate the role of *OsAHL* genes in the response to stress, based on promoter *cis*-element analysis. Our research will help identify potential *AHL* genes and provide a conceptual framework for further investigation of AHL gene activity in *O. sativa* under salt and drought stress. Some of the important OsAHL proteins interact with other proteins, which indirectly suggests that *AHL* genes also play very important roles in different plant growth and development activities and stress signaling. Finally, the functional validation of *AHL* genes was conducted by qRT-PCR in heat-contrasting genotypes of rice. The result clearly showed that most of the *AHL* family genes are involved in the drought and salt stress response pathways of rice. *OsAHL10*, *OsAHL13*, and *OsAHL20* genes showed expression under both salt and drought stress conditions, suggesting their role in the signaling of drought and salt stress. This study laid a foundation for further study on the function of the rice *AHL* gene family and also provided a theoretical basis for rice breeding.

## Data availability statement

The original contributions presented in the study are included in the article/**Supplementary Material**. Further inquiries can be directed to the corresponding author.

## Author contributions

AS: Conceptualization, Supervision, Writing – original draft. DA: Investigation, Writing – review & editing. DC: Data curation, Software, Writing – review & editing. RJ: Supervision, Writing – review & editing. SB: Investigation, Writing – review & editing. VS: Supervision, Writing – review & editing.
