# Supplementary material for: Genome-wide dissection of AT-hook motif nuclear-localized gene family and their expression profiling for drought and salt stress in rice (Oryza sativa)
Source: Front Plant Sci. 2023 Dec 12;14:1283555. doi: 10.3389/fpls.2023.1283555 (PMC10749976; doi:10.3389/fpls.2023.1283555)
Supplement: Supplementary file 1 [file DataSheet_1.docx]

**Supplementary Table 1:** List of primer and their sequences for qRT-PCR

| **Sr. No.** | **Product size** | **Locus ID** | **Sequences** | **F/R** | **Tm** | **G+C** | **Length bp** |
| --- | --- | --- | --- | --- | --- | --- | --- |
|  |  |  |  |  | **(^0^C)** | **(%)** |  |
| 1 | 58 | *OsAHL1* | TGACTTATGAGGGCCGGTTT | F | 59 | 50 | 20 |
|  |  |  | TCAGTTGGCGTGAATGATCC | R | 58.3 | 50 | 20 |
| 2 | 65 | *OsAHL2* | GCGACTTCCATCACCATCTC | F | 58.4 | 55 | 20 |
|  |  |  | TTGGTCATCCTCGGTCTTGA | R | 58.4 | 50 | 20 |
| 3 | 85 | *OsAHL3* | GGGAATGTGGGAGGCTATCA | F | 58.9 | 55 | 20 |
|  |  |  | AGAACGGTGGCCTGACTC | R | 58.9 | 61.1 | 18 |
| 4 | 110 | *OsAHL4* | ACCACGCCGACGAGAACAACGAAT | F | 64.4 | 54.2 | 24 |
|  |  |  | TGAGCCCTCCTCTTCTTCCTCCAAT | R | 64.4 | 52.3 | 25 |
| 5 | 55 | *OsAHL5* | ACTCTGGTGGTGTGGTTACA | F | 58.5 | 50 | 20 |
|  |  |  | CCAGACAGGCACAGGATTTC | R | 58.5 | 55 | 20 |
| 6 | 123 | *OsAHL6* | CCTGTCCAGGTAGTTGTTGC | F | 58.5 | 55 | 20 |
|  |  |  | ACTAGACAGAGTGCTGCCAT | R | 58.4 | 50 | 20 |
| 7 | 122 | *OsAHL7* | GCACTGGCACCGGTTAATCAATGT | F | 62.7 | 50 | 24 |
|  |  |  | ATTGAGCAGTGCATGCAGGGAGTA | R | 62.7 | 50 | 24 |
| 8 | 147 | *OsAHL8* | GCATTTTCACAACAAGGCCC | F | 58.5 | 50 | 20 |
|  |  |  | AAATGAGCCCGACAGAGAGA | R | 58.4 | 50 | 20 |
| 9 | 138 | *OsAHL9* | GTTCTTCAACCTGCCGCTCAACAACA | F | 64.8 | 50 | 26 |
|  |  |  | ATCGATCGGTGGTAACCCTGAATACG | R | 64.8 | 50 | 26 |
| 10 | 62 | *OsAHL10* | CACCGTTCAGGTGGTACTTG | F | 58.5 | 55 | 20 |
|  |  |  | CCTTCTTCGACTTGGGCTTC | R | 58.6 | 55 | 20 |
| 11 | 98 | *OsAHL11* | AGACCATCTGCCACACAACCTGAT | F | 62.7 | 50 | 24 |
|  |  |  | AACCATCCAACCATCCTTAGCTGCTG | R | 64.8 | 50 | 26 |
| 12 | 51 | *OsAHL12* | CCTGTTCAGGTGGTCATTGG | F | 58.5 | 55 | 20 |
|  |  |  | GTGCCCCTTCTTCACATCAG | R | 58.5 | 55 | 20 |
| 13 | 52 | *OsAHL13* | GCATGGGTGTAAAGGAGGAAA | F | 58.2 | 47.6 | 21 |
|  |  |  | CATGTCTGATACCCCACAAACA | R | 58.3 | 45.5 | 22 |
| 14 | 103 | *OsAHL14* | ACACTGCGTCAACCTGATTC | F | 58.5 | 50 | 20 |
|  |  |  | TTCCGCTGTTTTCAGTAGGC | R | 58.5 | 50 | 20 |
| 15 | 128 | *OsAHL15* | GCATGCCACCATTAGGCGTTGAT | F | 62.4 | 52.2 | 23 |
|  |  |  | CATTCGAAGCAGCAAAGACACCTGAC | R | 64.8 | 50 | 26 |
| 16 | 78 | *OsAHL16* | CACTTCGCCAAACTGCTACT | F | 58.5 | 50 | 20 |
|  |  |  | AAGAGCCAGACAGCGATAGT | R | 58.5 | 50 | 20 |
| 17 | 135 | *OsAHL17* | TCATGGTGATCGCCTCCACCTTC | F | 64.2 | 56.5 | 23 |
|  |  |  | TATATGGCCCTGGCACTTGAACCA | R | 62.7 | 50 | 24 |
| 18 | 77 |  | AGCGAATGGTGCAATAAGCA | F | 58.5 | 45 | 20 |
|  |  | *OsAHL18* | ATCGGCCCTCATAAGTCACA | R | 58.5 | 50 | 20 |
| 19 | 139 |  | ACCCCTCATGTCATCATCGT | F | 58.5 | 50 | 20 |
|  |  | *OsAHL19* | ATCCCAGCTACTACACCACC | R | 58.5 | 55 | 20 |
| 20 | 69 |  | GAAAGGGGGAATGGTATGCG | F | 58.7 | 55 | 20 |
|  |  | *OsAHL20* | CTGTGGCGACAATGATGACA | R | 58.6 | 50 | 20 |

**Supplementary Table 2**: The detail Promoter cis-element information of AHL genes in O.sativa.

| **Gene ID** | **cis-elements** | **Sequence** | **Description** |
| --- | --- | --- | --- |
| OsAHL1 | GARE-motif | TCTGTTG | gibberellin-responsive element |
| OsAHL1 | GARE | CGTCA | cis-acting regulatory element involved in the MeJA-responsiveness |
| OsAHL1 | TGACG-motif | TGACG | cis-acting regulatory element involved in the MeJA-responsiveness |
| OsAHL1 | ABRE | ACGTG | cis-acting element involved in the abscisic acid responsiveness |
| OsAHL1 | P-box | CCTTTTG | gibberellin-responsive element |
| OsAHL2 | SARE | TTCGACCATCTT | cis-acting element involved in salicylic acid responsiveness |
| OsAHL2 | MBS | CAACTG | MYB binding site involved in drought-inducibility |
| OsAHL2 | MBS | CAACTG | MYB binding site involved in drought-inducibility |
| OsAHL2 | MBS | CAACTG | MYB binding site involved in drought-inducibility |
| OsAHL2 | TC-rich repeats | GTTTTCTTAC | cis-acting element involved in defense and stress responsiveness |
| OsAHL2 | ABRE | ACGTG | cis-acting element involved in the abscisic acid responsiveness |
| OsAHL2 | ABRE | GACACGTACGT | cis-acting element involved in the abscisic acid responsiveness |
| OsAHL2 | TGACG-motif | TGACG | cis-acting regulatory element involved in the MeJA-responsiveness |
| OsAHL2 | CGTCA-motif | CGTCA | cis-acting regulatory element involved in the MeJA-responsiveness |
| OsAHL3 | MBS | CAACTG | MYB binding site involved in drought-inducibility |
| OsAHL3 | LTR | CCGAAA | cis-acting element involved in low-temperature responsiveness |
| OsAHL3 | LTR | CCGAAA | cis-acting element involved in low-temperature responsiveness |
| OsAHL3 | TGA-element | AACGAC | auxin-responsive element |
| OsAHL3 | TGACG-motif | TGACG | cis-acting regulatory element involved in the MeJA-responsiveness |
| OsAHL3 | TGACG-motif | TGACG | cis-acting regulatory element involved in the MeJA-responsiveness |
| OsAHL3 | CGTCA-motif | CGTCA | cis-acting regulatory element involved in the MeJA-responsiveness |
| OsAHL3 | CGTCA-motif | CGTCA | cis-acting regulatory element involved in the MeJA-responsiveness |
| OsAHL4 | TCA-element | CCATCTTTTT | cis-acting element involved in salicylic acid responsiveness |
| OsAHL4 | AuxRR-core | GGTCCAT | cis-acting regulatory element involved in auxin responsiveness |
| OsAHL4 | TGACG-motif | TGACG | cis-acting regulatory element involved in the MeJA-responsiveness |
| OsAHL4 | TGACG-motif | TGACG | cis-acting regulatory element involved in the MeJA-responsiveness |
| OsAHL4 | CGTCA-motif | CGTCA | cis-acting regulatory element involved in the MeJA-responsiveness |
| OsAHL4 | CGTCA-motif | CGTCA | cis-acting regulatory element involved in the MeJA-responsiveness |
| OsAHL4 | ABRE | CGTACGTGCA | cis-acting element involved in the abscisic acid responsiveness |
| OsAHL4 | ABRE | TACGTGTC | cis-acting element involved in the abscisic acid responsiveness |
| OsAHL4 | ABRE | ACGTG | cis-acting element involved in the abscisic acid responsiveness |
| OsAHL4 | ABRE | ACGTG | cis-acting element involved in the abscisic acid responsiveness |
| OsAHL4 | ABRE | ACGTG | cis-acting element involved in the abscisic acid responsiveness |
| OsAHL4 | ABRE | ACGTG | cis-acting element involved in the abscisic acid responsiveness |
| OsAHL5 | LTR | CCGAAA | cis-acting element involved in low-temperature responsiveness |
| OsAHL5 | TCA-element | TCAGAAGAGG | cis-acting element involved in salicylic acid responsiveness |
| OsAHL5 | TCA-element | TCAGAAGAGG | cis-acting element involved in salicylic acid responsiveness |
| OsAHL5 | TCA-element | TCAGAAGAGG | cis-acting element involved in salicylic acid responsiveness |
| OsAHL6 | TC-rich repeats | ATTCTCTAAC | cis-acting element involved in defense and stress responsiveness |
| OsAHL6 | TC-rich repeats | ATTCTCTAAC | cis-acting element involved in defense and stress responsiveness |
| OsAHL6 | LTR | CCGAAA | cis-acting element involved in low-temperature responsiveness |
| OsAHL6 | CGTCA-motif | CGTCA | cis-acting regulatory element involved in the MeJA-responsiveness |
| OsAHL6 | CGTCA-motif | CGTCA | cis-acting regulatory element involved in the MeJA-responsiveness |
| OsAHL6 | CGTCA-motif | CGTCA | cis-acting regulatory element involved in the MeJA-responsiveness |
| OsAHL6 | CGTCA-motif | CGTCA | cis-acting regulatory element involved in the MeJA-responsiveness |
| OsAHL6 | CGTCA-motif | CGTCA | cis-acting regulatory element involved in the MeJA-responsiveness |
| OsAHL6 | CGTCA-motif | CGTCA | cis-acting regulatory element involved in the MeJA-responsiveness |
| OsAHL6 | TGACG-motif | TGACG | cis-acting regulatory element involved in the MeJA-responsiveness |
| OsAHL6 | TGACG-motif | TGACG | cis-acting regulatory element involved in the MeJA-responsiveness |
| OsAHL6 | TGACG-motif | TGACG | cis-acting regulatory element involved in the MeJA-responsiveness |
| OsAHL6 | TGACG-motif | TGACG | cis-acting regulatory element involved in the MeJA-responsiveness |
| OsAHL6 | TGACG-motif | TGACG | cis-acting regulatory element involved in the MeJA-responsiveness |
| OsAHL6 | TGACG-motif | TGACG | cis-acting regulatory element involved in the MeJA-responsiveness |
| OsAHL7 | TCA-element | CCATCTTTTT | cis-acting element involved in salicylic acid responsiveness |
| OsAHL7 | MBS | CAACTG | MYB binding site involved in drought-inducibility |
| OsAHL7 | TGA-element | AACGAC | auxin-responsive element |
| OsAHL7 | TGA-element | AACGAC | auxin-responsive element |
| OsAHL7 | ABRE | ACGTG | cis-acting element involved in the abscisic acid responsiveness |
| OsAHL7 | ABRE | ACGTG | cis-acting element involved in the abscisic acid responsiveness |
| OsAHL7 | ABRE | CGCACGTGTC | cis-acting element involved in the abscisic acid responsiveness |
| OsAHL7 | ABRE | CACGTG | cis-acting element involved in the abscisic acid responsiveness |
| OsAHL7 | ABRE | ACGTG | cis-acting element involved in the abscisic acid responsiveness |
| OsAHL7 | ABRE | CACGTG | cis-acting element involved in the abscisic acid responsiveness |
| OsAHL7 | ABRE | ACGTG | cis-acting element involved in the abscisic acid responsiveness |
| OsAHL7 | ABRE | CGTACGTGCA | cis-acting element involved in the abscisic acid responsiveness |
| OsAHL7 | ABRE | ACGTG | cis-acting element involved in the abscisic acid responsiveness |
| OsAHL7 | ABRE | ACGTG | cis-acting element involved in the abscisic acid responsiveness |
| OsAHL7 | TGACG-motif | TGACG | cis-acting regulatory element involved in the MeJA-responsiveness |
| OsAHL7 | CGTCA-motif | CGTCA | cis-acting regulatory element involved in the MeJA-responsiveness |
| OsAHL8 | TCA-element | CCATCTTTTT | cis-acting element involved in salicylic acid responsiveness |
| OsAHL8 | P-box | CCTTTTG | gibberellin-responsive element |
| OsAHL8 | MBS | CAACTG | MYB binding site involved in drought-inducibility |
| OsAHL8 | MBS | CAACTG | MYB binding site involved in drought-inducibility |
| OsAHL8 | GARE-motif | TCTGTTG | gibberellin-responsive element |
| OsAHL8 | TGACG-motif | TGACG | cis-acting regulatory element involved in the MeJA-responsiveness |
| OsAHL8 | CGTCA-motif | CGTCA | cis-acting regulatory element involved in the MeJA-responsiveness |
| OsAHL9 | MBS | CAACTG | MYB binding site involved in drought-inducibility |
| OsAHL9 | MBS | CAACTG | MYB binding site involved in drought-inducibility |
| OsAHL9 | TATC-box | TATCCCA | cis-acting element involved in gibberellin-responsiveness |
| OsAHL9 | LTR | CCGAAA | cis-acting element involved in low-temperature responsiveness |
| OsAHL9 | CGTCA-motif | CGTCA | cis-acting regulatory element involved in the MeJA-responsiveness |
| OsAHL9 | CGTCA-motif | CGTCA | cis-acting regulatory element involved in the MeJA-responsiveness |
| OsAHL9 | TGACG-motif | TGACG | cis-acting regulatory element involved in the MeJA-responsiveness |
| OsAHL9 | TGACG-motif | TGACG | cis-acting regulatory element involved in the MeJA-responsiveness |
| OsAHL10 | TATC-box | TATCCCA | cis-acting element involved in gibberellin-responsiveness |
| OsAHL10 | TCA-element | CCATCTTTTT | cis-acting element involved in salicylic acid responsiveness |
| OsAHL10 | TGACG-motif | TGACG | cis-acting regulatory element involved in the MeJA-responsiveness |
| OsAHL10 | TGACG-motif | TGACG | cis-acting regulatory element involved in the MeJA-responsiveness |
| OsAHL10 | CGTCA-motif | CGTCA | cis-acting regulatory element involved in the MeJA-responsiveness |
| OsAHL10 | CGTCA-motif | CGTCA | cis-acting regulatory element involved in the MeJA-responsiveness |
| OsAHL10 | ABRE | ACGTG | cis-acting element involved in the abscisic acid responsiveness |
| OsAHL10 | ABRE | ACGTG | cis-acting element involved in the abscisic acid responsiveness |
| OsAHL11 | CGTCA-motif | CGTCA | cis-acting regulatory element involved in the MeJA-responsiveness |
| OsAHL11 | CGTCA-motif | CGTCA | cis-acting regulatory element involved in the MeJA-responsiveness |
| OsAHL11 | TGACG-motif | TGACG | cis-acting regulatory element involved in the MeJA-responsiveness |
| OsAHL11 | TGACG-motif | TGACG | cis-acting regulatory element involved in the MeJA-responsiveness |
| OsAHL11 | ABRE | CACGTG | cis-acting element involved in the abscisic acid responsiveness |
| OsAHL11 | ABRE | ACGTG | cis-acting element involved in the abscisic acid responsiveness |
| OsAHL11 | ABRE | GCAACGTGTC | cis-acting element involved in the abscisic acid responsiveness |
| OsAHL11 | ABRE | ACGTG | cis-acting element involved in the abscisic acid responsiveness |
| OsAHL11 | ABRE | ACGTG | cis-acting element involved in the abscisic acid responsiveness |
| OsAHL11 | ABRE | ACGTG | cis-acting element involved in the abscisic acid responsiveness |
| OsAHL12 | TGA-element | AACGAC | auxin-responsive element |
| OsAHL12 | TGA-element | AACGAC | auxin-responsive element |
| OsAHL12 | ABRE | ACGTG | cis-acting element involved in the abscisic acid responsiveness |
| OsAHL12 | ABRE | CACGTG | cis-acting element involved in the abscisic acid responsiveness |
| OsAHL12 | ABRE | ACGTG | cis-acting element involved in the abscisic acid responsiveness |
| OsAHL12 | TGACG-motif | TGACG | cis-acting regulatory element involved in the MeJA-responsiveness |
| OsAHL12 | TGACG-motif | TGACG | cis-acting regulatory element involved in the MeJA-responsiveness |
| OsAHL12 | CGTCA-motif | CGTCA | cis-acting regulatory element involved in the MeJA-responsiveness |
| OsAHL12 | CGTCA-motif | CGTCA | cis-acting regulatory element involved in the MeJA-responsiveness |
| OsAHL12 | MBS | CAACTG | MYB binding site involved in drought-inducibility |
| OsAHL12 | MBS | CAACTG | MYB binding site involved in drought-inducibility |
| OsAHL13 | ABRE | ACGTG | cis-acting element involved in the abscisic acid responsiveness |
| OsAHL13 | ABRE | ACGTG | cis-acting element involved in the abscisic acid responsiveness |
| OsAHL13 | ABRE | ACGTG | cis-acting element involved in the abscisic acid responsiveness |
| OsAHL13 | TGACG-motif | TGACG | cis-acting regulatory element involved in the MeJA-responsiveness |
| OsAHL13 | CGTCA-motif | CGTCA | cis-acting regulatory element involved in the MeJA-responsiveness |
| OsAHL13 | AuxRR-core | GGTCCAT | cis-acting regulatory element involved in auxin responsiveness |
| OsAHL13 | MBS | CAACTG | MYB binding site involved in drought-inducibility |
| OsAHL14 | P-box | CCTTTTG | gibberellin-responsive element |
| OsAHL14 | TGACG-motif | TGACG | cis-acting regulatory element involved in the MeJA-responsiveness |
| OsAHL14 | TGACG-motif | TGACG | cis-acting regulatory element involved in the MeJA-responsiveness |
| OsAHL14 | CGTCA-motif | CGTCA | cis-acting regulatory element involved in the MeJA-responsiveness |
| OsAHL14 | CGTCA-motif | CGTCA | cis-acting regulatory element involved in the MeJA-responsiveness |
| OsAHL14 | ABRE | ACGTG | cis-acting element involved in the abscisic acid responsiveness |
| OsAHL14 | ABRE | TACGTGTC | cis-acting element involved in the abscisic acid responsiveness |
| OsAHL14 | ABRE | ACGTG | cis-acting element involved in the abscisic acid responsiveness |
| OsAHL14 | TGA-element | AACGAC | auxin-responsive element |
| OsAHL14 | LTR | CCGAAA | cis-acting element involved in low-temperature responsiveness |
| OsAHL15 | ABRE | ACGTG | cis-acting element involved in the abscisic acid responsiveness |
| OsAHL15 | ABRE | CGTACGTGCA | cis-acting element involved in the abscisic acid responsiveness |
| OsAHL15 | ABRE | ACGTG | cis-acting element involved in the abscisic acid responsiveness |
| OsAHL15 | AuxRR-core | GGTCCAT | cis-acting regulatory element involved in auxin responsiveness |
| OsAHL15 | TC-rich repeats | GTTTTCTTAC | cis-acting element involved in defense and stress responsiveness |
| OsAHL15 | TC-rich repeats | GTTTTCTTAC | cis-acting element involved in defense and stress responsiveness |
| OsAHL16 | CGTCA-motif | CGTCA | cis-acting regulatory element involved in the MeJA-responsiveness |
| OsAHL16 | CGTCA-motif | CGTCA | cis-acting regulatory element involved in the MeJA-responsiveness |
| OsAHL16 | TGACG-motif | TGACG | cis-acting regulatory element involved in the MeJA-responsiveness |
| OsAHL16 | TGACG-motif | TGACG | cis-acting regulatory element involved in the MeJA-responsiveness |
| OsAHL16 | ABRE | ACGTG | cis-acting element involved in the abscisic acid responsiveness |
| OsAHL16 | ABRE | AACCCGG | cis-acting element involved in the abscisic acid responsiveness |
| OsAHL16 | ABRE | ACGTG | cis-acting element involved in the abscisic acid responsiveness |
| OsAHL16 | MBS | CAACTG | MYB binding site involved in drought-inducibility |
| OsAHL16 | P-box | CCTTTTG | gibberellin-responsive element |
| OsAHL16 | P-box | CCTTTTG | gibberellin-responsive element |
| OsAHL16 | P-box | CCTTTTG | gibberellin-responsive element |
| OsAHL17 | TATC-box | TATCCCA | cis-acting element involved in gibberellin-responsiveness |
| OsAHL17 | TGA-element | AACGAC | auxin-responsive element |
| OsAHL17 | TC-rich repeats | ATTCTCTAAC | cis-acting element involved in defense and stress responsiveness |
| OsAHL18 | AuxRR-core | GGTCCAT | cis-acting regulatory element involved in auxin responsiveness |
| OsAHL18 | TGACG-motif | TGACG | cis-acting regulatory element involved in the MeJA-responsiveness |
| OsAHL18 | CGTCA-motif | CGTCA | cis-acting regulatory element involved in the MeJA-responsiveness |
| OsAHL18 | ABRE | GACACGTACGT | cis-acting element involved in the abscisic acid responsiveness |
| OsAHL18 | ABRE | ACGTG | cis-acting element involved in the abscisic acid responsiveness |
| OsAHL18 | ABRE | CACGTG | cis-acting element involved in the abscisic acid responsiveness |
| OsAHL18 | ABRE | ACGTG | cis-acting element involved in the abscisic acid responsiveness |
| OsAHL18 | MBS | CAACTG | MYB binding site involved in drought-inducibility |
| OsAHL18 | MBS | CAACTG | MYB binding site involved in drought-inducibility |
| OsAHL18 | TCA-element | CCATCTTTTT | cis-acting element involved in salicylic acid responsiveness |
| OsAHL19 | MBS | CAACTG | MYB binding site involved in drought-inducibility |
| OsAHL19 | CGTCA-motif | CGTCA | cis-acting regulatory element involved in the MeJA-responsiveness |
| OsAHL19 | TGACG-motif | TGACG | cis-acting regulatory element involved in the MeJA-responsiveness |
| OsAHL19 | ABRE | TACGTGTC | cis-acting element involved in the abscisic acid responsiveness |
| OsAHL19 | ABRE | ACGTG | cis-acting element involved in the abscisic acid responsiveness |
| OsAHL19 | ABRE | ACGTG | cis-acting element involved in the abscisic acid responsiveness |
| OsAHL19 | LTR | CCGAAA | cis-acting element involved in low-temperature responsiveness |
| OsAHL19 | LTR | CCGAAA | cis-acting element involved in low-temperature responsiveness |
| OsAHL20 | ABRE | ACGTG | cis-acting element involved in the abscisic acid responsiveness |
| OsAHL20 | ABRE | TACGGTC | cis-acting element involved in the abscisic acid responsiveness |

**Supplementary Table 3:** Physical and chemical properties, of 20 AHL groups of genes in rice

| **Locus IDs** | ***AHL* gene in rice** | **Amino acid** | **Mol. Weight** | **PI** | **Instability Index** | **Alipathic Index** | **Hydropathicity (G*RAV*Y)** |
| --- | --- | --- | --- | --- | --- | --- | --- |
| LOC_Os02g03270 | *OsAHL1* | 388 | 38533.26 | 9.09 | 47.66 | 74.33 | -0. 033 |
| LOC_Os02g25020 | *OsAHL2* | 316 | 32170.76 | 6.03 | 46.66 | 75.09 | -0. 309 |
| LOC_Os02g48320 | *OsAHL3* | 336 | 32669. 37 | 6.71 | 60.38 | 66.49 | -0.204 |
| LOC_Os02g57520 | *OsAHL4* | 266 | 26625.91 | 5.76 | 53.88 | 70.15 | -0.236 |
| LOC_Os02g57820 | *OsAHL5* | 394 | 40028.53 | 6.97 | 45.14 | 60.41 | -0.372 |
| LOC_Os03g01540 | *OsAHL6* | 298 | 31675.44 | 8.43 | 58.05 | 91.91 | -0.164 |
| LOC_Os03g16350 | *OsAHL7* | 258 | 25858.91 | 6.6 | 56.17 | 67.4 | -0.219 |
| LOC_Os04g49990 | *OsAHL8* | 379 | 38240.73 | 9.06 | 50.13 | 53.11 | -0.479 |
| LOC_Os04g50030 | *OsAHL9* | 305 | 30691.22 | 6.18 | 56.25 | 71.38 | -0.248 |
| LOC_Os04g58730 | *OsAHL10* | 420 | 42840.10 | 9.8 | 66 | 59.9 | -0.423 |
| LOC_Os06g04540 | *OsAHL11* | 328 | 33416.26 | 6.56 | 59.29 | 71.77 | -0.273 |
| LOC_Os06g22030 | *OsAHL12* | 378 | 37450.18 | 10.46 | 53.86 | 66.75 | -0.224 |
| LOC_Os07g13100 | *OsAHL13* | 201 | 20510.56 | 6.96 | 52.7 | 80.2 | 0.129 |
| LOC_Os08g02490 | *OsAHL14* | 372 | 37813.56 | 9.42 | 45.43 | 72.69 | -0.12 |
| LOC_Os08g06320 | *OsAHL15* | 289 | 28298.74 | 6.29 | 62.48 | 74.36 | -0.045 |
| LOC_Os08g40150 | *OsAHL16* | 354 | 35262.8 | 10.08 | 50.54 | 65.68 | -0.227 |
| LOC_Os08g44910 | *OsAHL17* | 235 | 23940.15 | 7.99 | 54.76 | 70.68 | -0.2 |
| LOC_Os10g42230 | *OsAHL18* | 405 | 41398.9 | 9.51 | 66.07 | 58.25 | -0.402 |
| LOC_Os11g05160 | *OsAHL19* | 366 | 37243.81 | 7.22 | 66.87 | 62.21 | -0.225 |
| LOC_Os12g05200 | *OsAHL20* | 280 | 29392.51 | 9.82 | 69.67 | 74.25 | -0.176 |

**Supplementary Table 4:** Localization, gene sequence length, and domain location of 20 AHL groups of genes in rice

| **Sr.NO.** | **Locus Id’s** | **Chromosomal location** | **WoLF** | **Location** | **Gen Sequence length (bp)** | **Domain (from -to)** | **E-value** |
| --- | --- | --- | --- | --- | --- | --- | --- |
|  |  |  | **PSORT** |  |  |  |  |
| 1 | LOC_Os02g03270 | 2 | E.R.:3.5, vacu: 3, E.R._plas: 2.5, nucl: 2, cyto_pero: 2, cyto: 1.5, pero: 1.5, mito: 1, extr: 1 | Nucleus | 5122 | 172-288 | 3.50E-39 |
| 2 | LOC_Os02g25020 | 2 | chlo: 3, nucl: 3, E.R.: 2, cyto: 1, mito: 1, plas: 1, vacu: 1, pero: 1, golg: 1 | Chloroplast, Nucleus | 1634 | 125-240 | 1.10E-41 |
| 3 | LOC_Os02g48320 | 2 | nucl: 4.5, cyto_nucl: 3.83333, cyto_pero: 2.33333, chlo: 2, cyto: 2, vacu: 2, E.R.: 1.5, pero: 1.5, E.R._plas: 1.5 | Chloroplast, Nucleus | 2520 | 127-245 | 9.50E-37 |
| 4 | LOC_Os02g57520 | 2 | cyto: 3.5, E.R.: 3.5, E.R._plas: 3.5, plas: 2.5, cyto_pero: 2.5, vacu: 2, nucl: 1, mito: 1 | Nucleus | 1707 | 86-202 | 5.70E-40 |
| 5 | LOC_Os02g57820 | 2 | nucl: 14 | Cytoplasm | 4314 | 210-327 | 2.30E-36 |
| 6 | LOC_Os03g01540 | 3 | nucl: 3, vacu: 3, cyto: 2, E.R.: 2, golg: 2, chlo: 1, extr: 1 | Nucleus | 2689 | 103-211 | 1.20E-23 |
| 7 | LOC_Os03g16350 | 3 | nucl: 12, chlo: 1, mito: 1 | Nucleus | 1387 | 72-189 | 1.10E-37 |
| 8 | LOC_Os04g49990 | 4 | nucl: 13, cyto: 1 | Nucleus | 4116 | 170-287 | 4.10E-38 |
| 9 | LOC_Os04g50030 | 4 | nucl: 3, E.R.: 2.5, E.R._plas: 2.5, cyto: 2, mito: 2, plas: 1.5, chlo: 1, vacu: 1, pero: 1 | Nucleus | 1771 | 110-226 | 3.10E-39 |
| 10 | LOC_Os04g58730 | 4 | plas: 3, vacu: 3, nucl: 2.5, chlo: 2, cysk_nucl: 2, cyto: 1.5, cyto_pero: 1.5, mito: 1 | Chloroplast, Nucleus | 4374 | 218-342 | 2.80E-27 |
| 11 | LOC_Os06g04540 | 6 | E.R.: 4.5, cyto: 3.5, E.R._plas: 3.5, cyto_pero: 2.5, mito: 2, plas: 1.5, nucl: 1, vacu: 1 | Nucleus | 1335 | 139-255 | 6.60E-38 |
| 12 | LOC_Os06g22030 | 6 | chlo: 10, nucl: 4 | Nucleus | 5779 | 91-102 | 0.0064 |
| 13 | LOC_Os07g13100 | 7 | cyto: 8, chlo: 3, nucl: 2, mito: 1 | Chloroplast | 2000 | 46-140 | 8.20E-22 |
| 14 | LOC_Os08g02490 | 8 | chlo: 3, vacu: 3, plas: 2.5, E.R._plas: 2.5, cyto: 1.5, E.R.: 1.5, cyto_pero: 1.5, mito: 1, extr: 1 | Nucleus | 3849 | 168-284 | 8.10E-44 |
| 15 | LOC_Os08g06320 | 8 | cyto: 3.5, cyto_nucl: 3.5, E.R.: 3, nucl: 2.5, pero: 2, mito: 1, vacu: 1, golg: 1 | Chloroplast | 1474 | 91-212 | 1.60E-39 |
| 16 | LOC_Os08g40150 | 8 | nucl: 3, cyto: 2.5, E.R.: 2.5, E.R._plas: 2.5, mito: 2, vacu: 2, cyto_pero: 2, plas: 1.5cysk: 1 | Nucleus | 4202 | 166-284 | 1.50E-41 |
| 17 | LOC_Os08g44910 | 8 | nucl: 9, chlo: 2, cyto: 2, plas: 1 | Chloroplast, Nucleus | 1698 | 63-179 | 2.00E-39 |
| 18 | LOC_Os10g42230 | 10 | plas: 3.5, E.R._plas: 2.5, chlo: 2, extr: 2, vacu: 2, cyto: 1.5, cyto_pero: 1.5, nucl: 1, mito: 1 | Nucleus | 4233 | 188-305 | 3.10E-42 |
| 19 | LOC_Os11g05160 | 11 | nucl: 12, cyto: 1, plas: 1 | Nucleus | 3078 | 137-252 | 2.20E-38 |
| 20 | LOC_Os12g05200 | 12 | chlo: 7, nucl: 5, cyto: 1, extr: 1 | Nucleus | 4181 | 47-162 | 3.40E-37 |


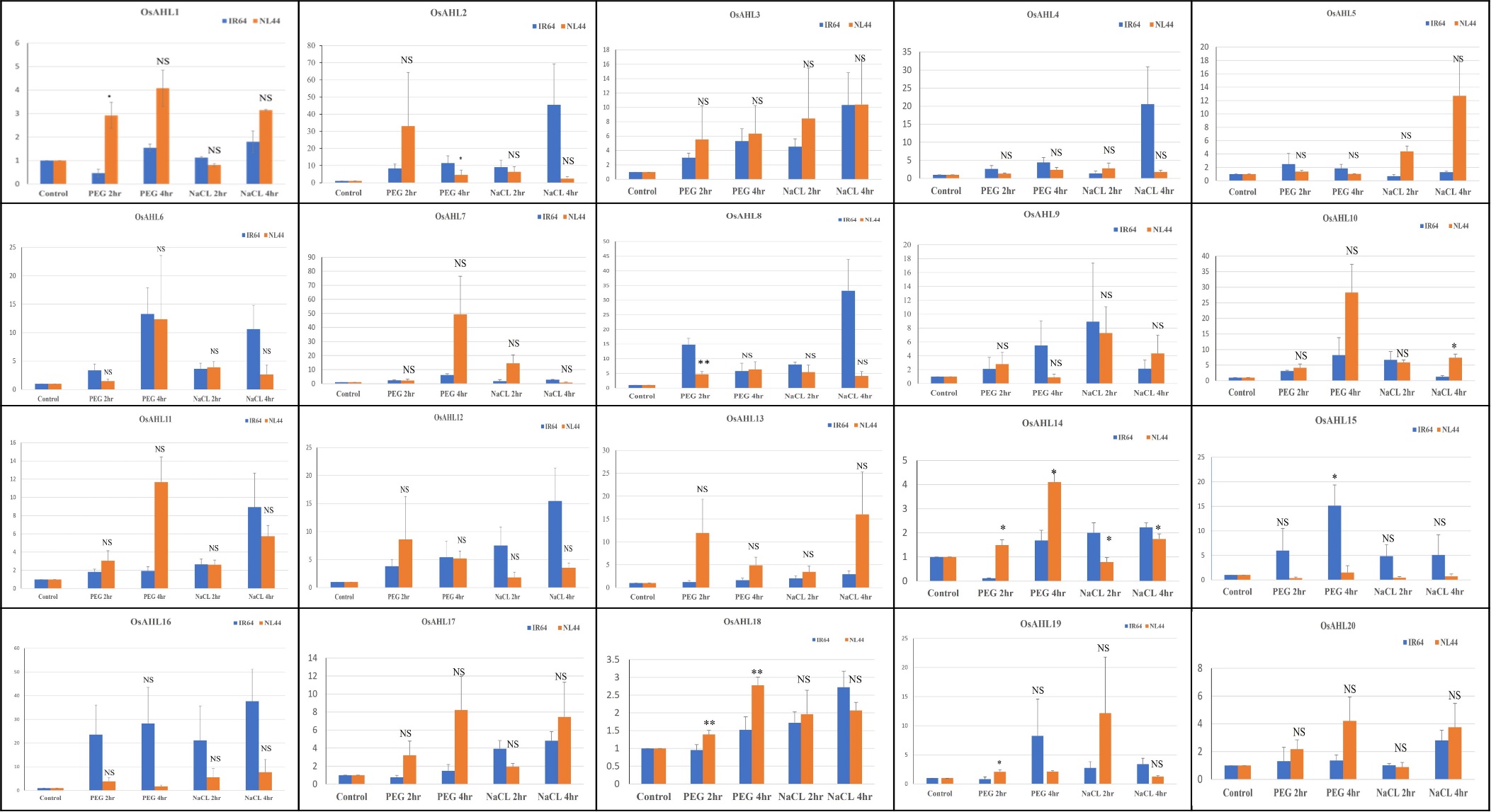


**Supplementary Figure 1:** Quantitative real-time PCR-based expression analysis of AHL genes in rice under drought and salinity stresses.

Error bars indicate the mean value of replicates ± SE. Asterisks over bars represent the results of the One-way ANOVA test indicating significant differences in expression levels as compared to control where *P < 0.05; **P < 0.01; are the significant differences, and NS means non-significant differences.
